# Supplementary material for: Development of Hydrophobic Cell-Penetrating Stapled Peptides as Drug Carriers
Source: Int J Mol Sci. 2023 Jul 21;24(14):11768. doi: 10.3390/ijms241411768 (PMC10380766; doi:10.3390/ijms241411768)
Supplement: Supplementary file 1 [file ijms-24-11768-s001.zip › ijms-2491543-supplementary.pdf]

Supporting information

Development of hydrophobic cell-penetrating stapled peptides as drug carriers

Keisuke Tsuchiya[1,2], Kanako Horikoshi[1,3], Minami Fujita[1,3], Motoharu Hirano[1,3], Maho Miyamoto[1,3], Hidetomo Yokoo[1,*], and Yosuke Demizu[1,3,4,*]

[1] *Division of Organic Chemistry, National Institute of Health Sciences, Kanagawa 210-9501, Japan; k.tsuchiya@rs.socu.ac.jp (K.T.); s202095f@yokohama-cu.ac.jp (K.H.); w225425e@yokohama-cu.ac.jp (M.F.); w225510f@yokohama-cu.ac.jp (M.H.); s202107d@yokohama-cu.ac.jp (M.M.)*

[2] *Division of Pharmaceutical Organic Chemistry, Faculty of Pharmaceutical Sciences, Sanyo-Onoda City University, 1-1-1 Daigakudori, Sanyo-Onoda-shi, Yamaguchi 756-0884, Japan*

[3] *Graduate School of Medical Life Science, Yokohama City University, 1-7-29 Suehiro-cho, Tsurumi-ku, Yokohama 230-0045, Japan*

[4] *Graduate School of Medicine, Dentistry and Pharmaceutical Sciences, Okayama University, 1-1-1 Tsushimanaka, Kita-ku, Okayama 700-8530, Japan*

* *Correspondence: e-mail: yokoo@nihs.go.jp (H.Y.), demizu@nihs.go.jp (Y.D.)*

Table of contents

Characterization of synthesized peptides S3

Figure S1. Cellular uptake of peptide/Cy5-pDNA complexes S7

Figure S2. Physicochemical characterization of peptide/pDNA complexes S7

Figure S3. Raw data for scatter plots and histograms in flow cytometry in Figure 3 S8−15

Figure S4. Raw data for scatter plots and histograms in flow cytometry in Figure 4a S16−31

Figure S5. Raw data for scatter plots and histograms in flow cytometry in Figure 4b S32−37

Figure S6. Raw data for scatter plots and histograms in flow cytometry in Figure 5 S38−43

Characterization of synthesized peptides

F-1 (CF-βAla-Ala-Gly-Tyr-Leu-Leu-Gly-Lys-Ile-Asn-Leu-Lys-Ala-Leu-Ala-Ala-Leu-Ala-Lys-Lys-Ile-Leu-NH_2)

LC/MS (ESI$^+$) calcd for $C_{128}H_{200}N_{28}O_{30}$ $[M+5H]^{5+}$: 523.1077; found : 522.8900

Purity : >99% (Rt = 21.1 min)

HPLC: Conditions = solvent A 0.1% TFA in water, solvent B 0.1% TFA in MeCN

Gradient = 10-90% gradient of solvent B over 30 min

Column: InertSustainSwift C18 column (3 µm HP, 4.6×250 mm, GL Sciences)

Flowrate: 1.0 mL/min

Temperature: 40 °C

Detection: 220 nm

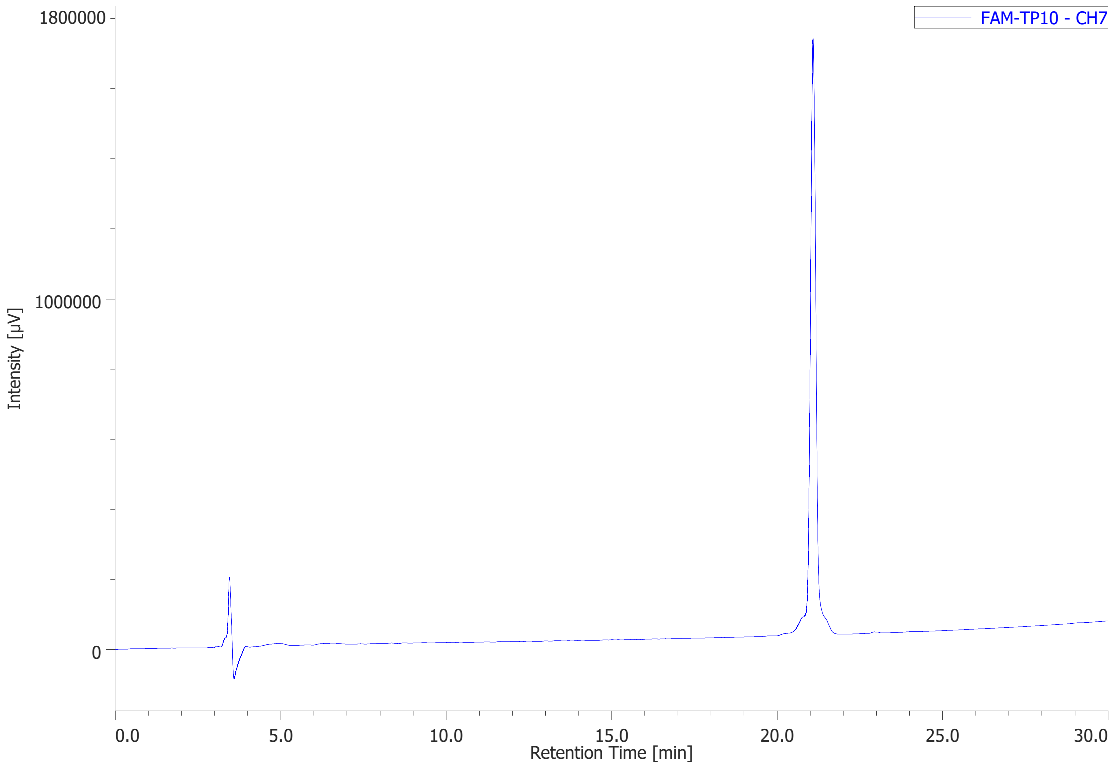

Analytical HPLC of **F-1**

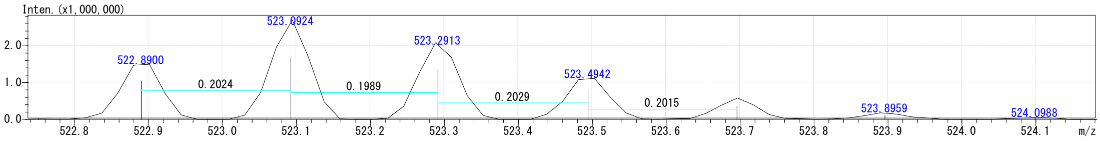

LC-MS analysis of peptide **F-1**

F-2 (CF-βAla-Ala-S_5^*-Tyr-Leu-Leu-S_5^*-Lys-Ile-Asn-Leu-Lys-Ala-Leu-Ala-Ala-Leu-Ala-Lys-Lys-Ile-Leu-NH_2)

LC/MS (ESI$^+$) calcd for $C_{138}H_{216}N_{28}O_{30}$ $[M+5H]^{5+}$: 550.3327; found : 550.1150

Purity : >99% (Rt = 25.7 min)

HPLC: Conditions = solvent A 0.1% TFA in water, solvent B 0.1% TFA in MeCN

Gradient = 10-90% gradient of solvent B over 30 min

Column: InertSustainSwift C18 column (3 µm HP, 4.6×250 mm, GL Sciences)

Flowrate: 1.0 mL/min

Temperature: 40 °C

Detection: 220 nm

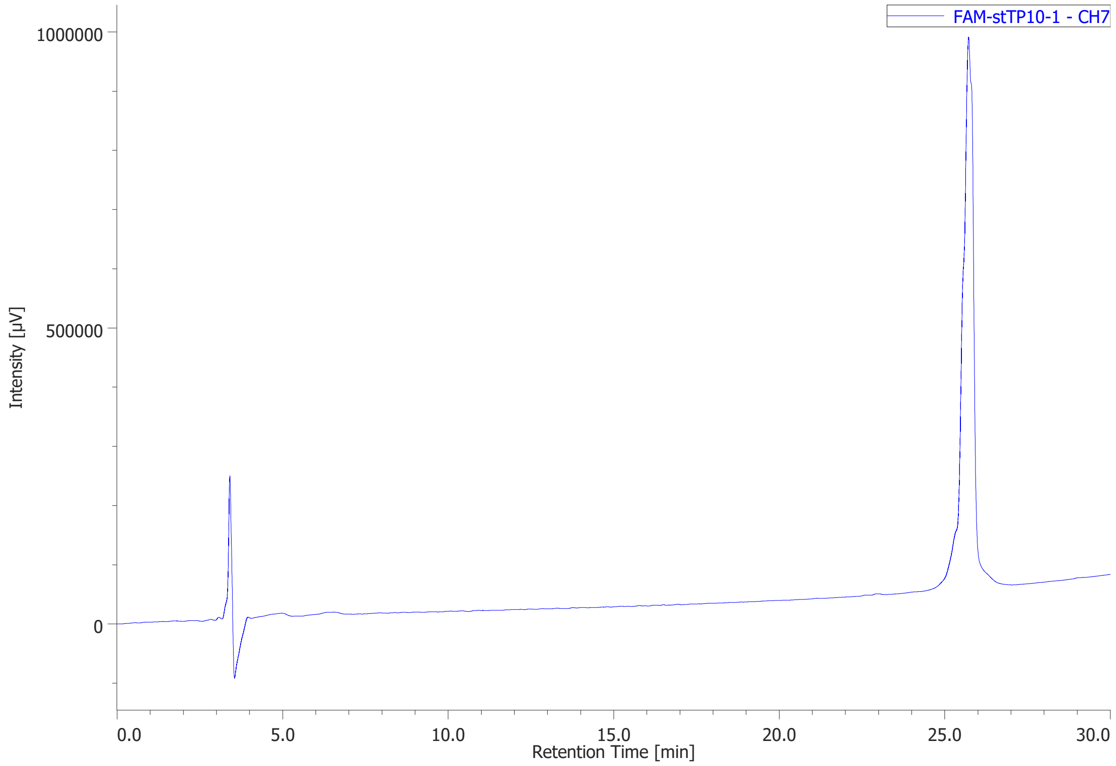

Analytical HPLC of **F-2**

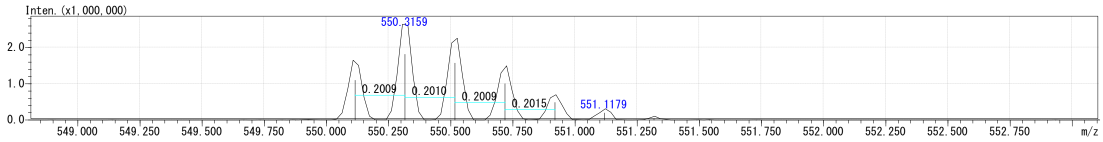

LC-MS analysis of peptide **F-2**

F-3 (CF-βAla-Ala-Gly-Tyr-Leu-Leu-Gly-Lys-Ile-Asn-Leu-Lys-Ala-Leu-Ala-Ala-S_5^*-Ala-Lys-Lys-S_5^*-Leu-NH_2)

LC/MS (ESI^+) calcd for $C_{130}H_{200}N_{28}O_{30}$ $[M+5H]^{5+}$: 527.9077; found : 527.6910

Purity : >99% (Rt = 21.2 min)

HPLC: Conditions = solvent A 0.1% TFA in water, solvent B 0.1% TFA in MeCN

Gradient = 10-90% gradient of solvent B over 30 min

Column: InertSustainSwift C18 column (3 µm HP, 4.6×250 mm, GL Sciences)

Flowrate: 1.0 mL/min

Temperature: 40 °C

Detection: 220 nm

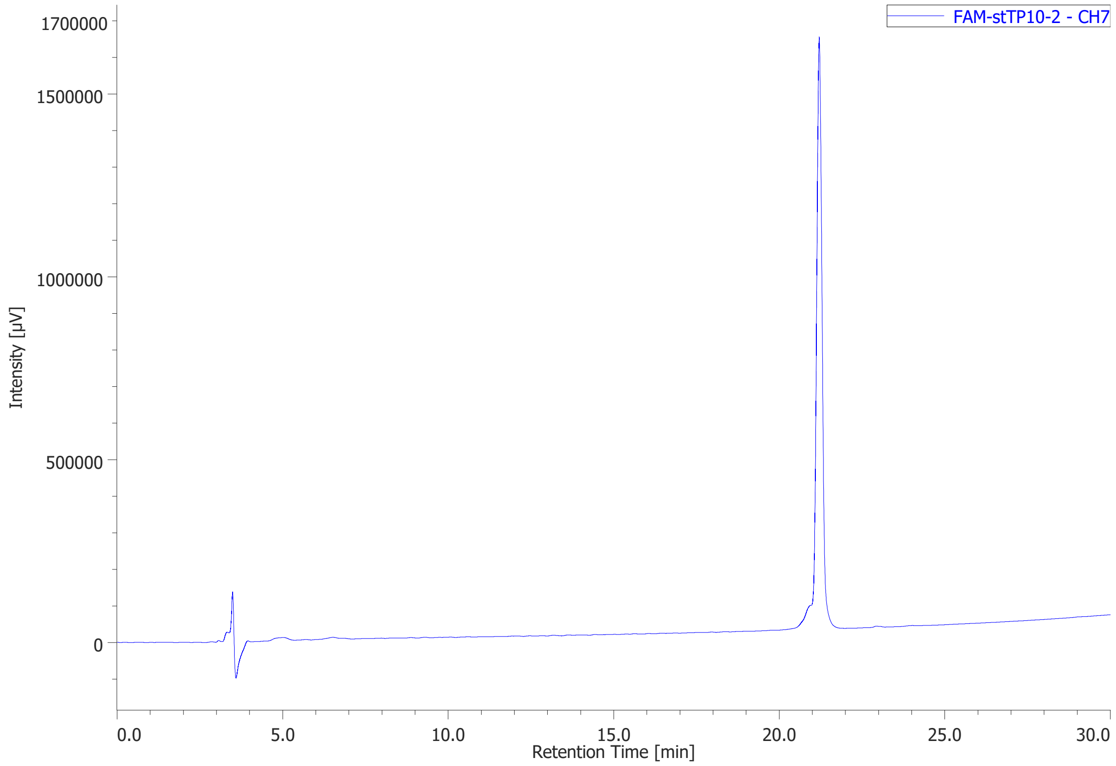

Analytical HPLC of **F-3**

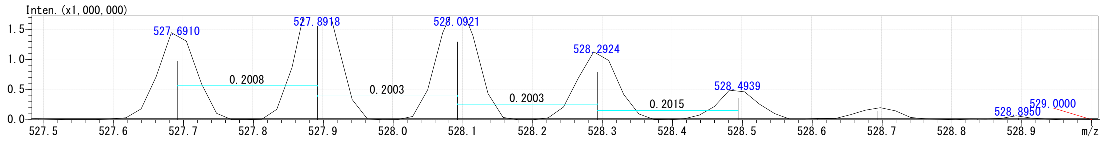

LC-MS analysis of peptide **F-3**

F-4 (CF-βAla-Ala-Gly-Tyr-S_5^*-Leu-Gly-Lys-S_5^*-Asn-Leu-Lys-Ala-Leu-Ala-Ala-Leu-Ala-Lys-Lys-Ile-Leu-NH_2)

LC/MS (ESI^+) calcd for $C_{130}H_{200}N_{28}O_{30}$ $[M+5H]^{5+}$: 527.9077; found : 527.7045

Purity : >99% (Rt = 20.7 min)

HPLC: Conditions = solvent A 0.1% TFA in water, solvent B 0.1% TFA in MeCN

Gradient = 10-90% gradient of solvent B over 30 min

Column: Inertsil WP300 C18 column (250 mm×4.6 mm, 5 μm, GL Sciences)

Flowrate: 1.0 mL/min

Temperature: 40 °C

Detection: 220 nm

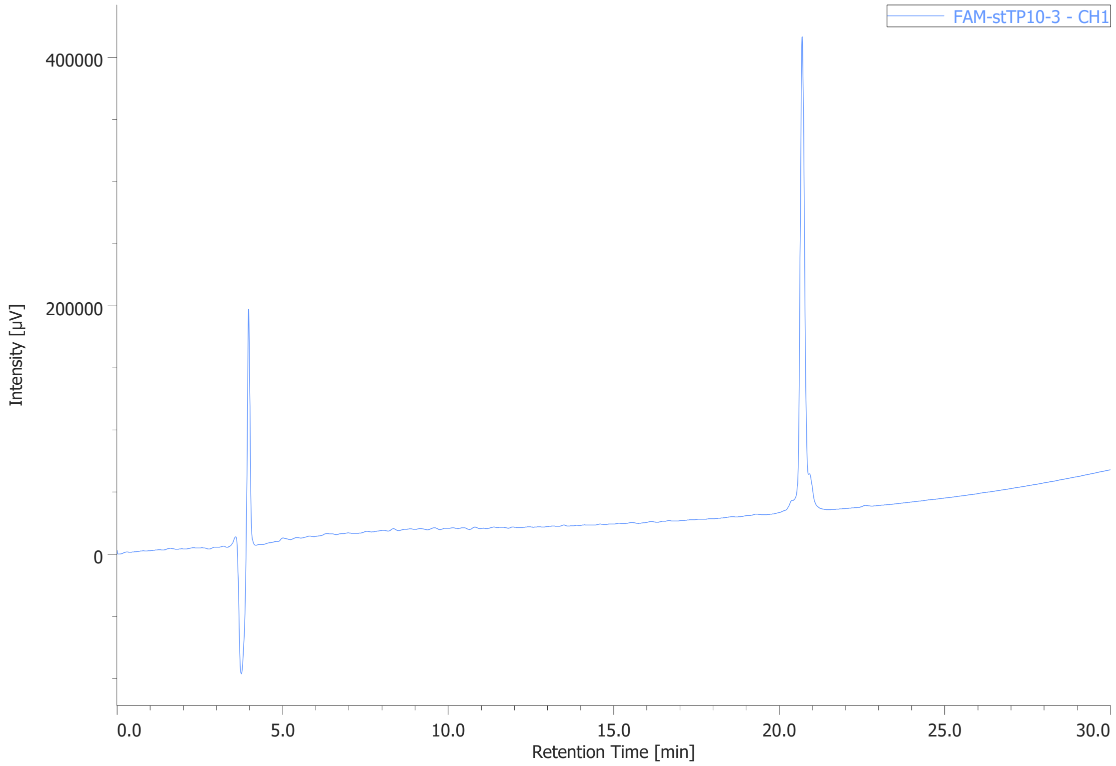

Analytical HPLC of **F-4**

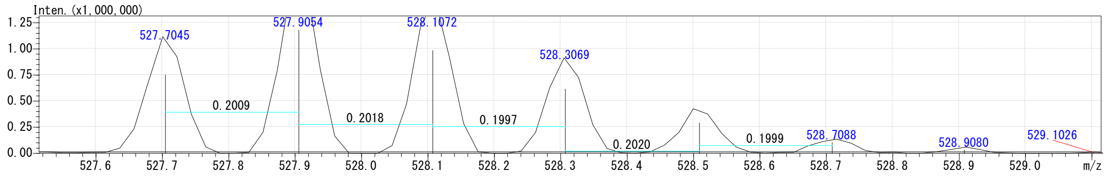

LC-MS analysis of peptide **F-4**

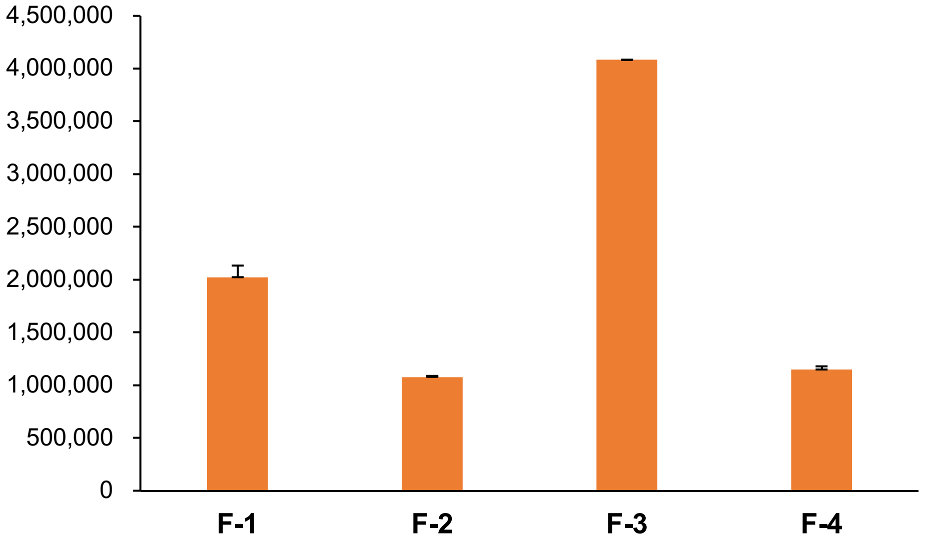

Figure S1. Cellular uptake of peptide/Cy5-pDNA complexes at N/P = 8 with HEK293 cells. Cellular uptake ability was determined by flow cytometry. The cells were excited with a 488 nm light from an LD laser for detecting FAM-peptide. Error bars represent the mean ± standard deviation, n = 4.

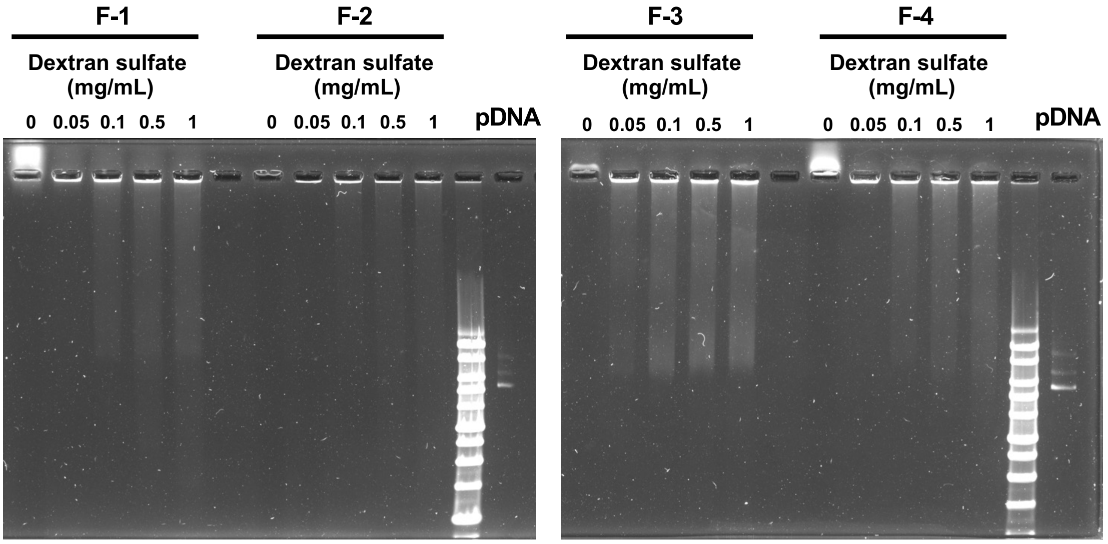

Figure S2. Physicochemical characterization of peptide/pDNA complexes at N/P = 8 with dextran sulfate. Gel retardation assay for evaluation of the stability and the dissociation of the peptide/pDNA complexes upon treatment with dextran sulfate at different concentrations ranging from 0 to 1 mg/mL.

(a) **F-1** 2 h

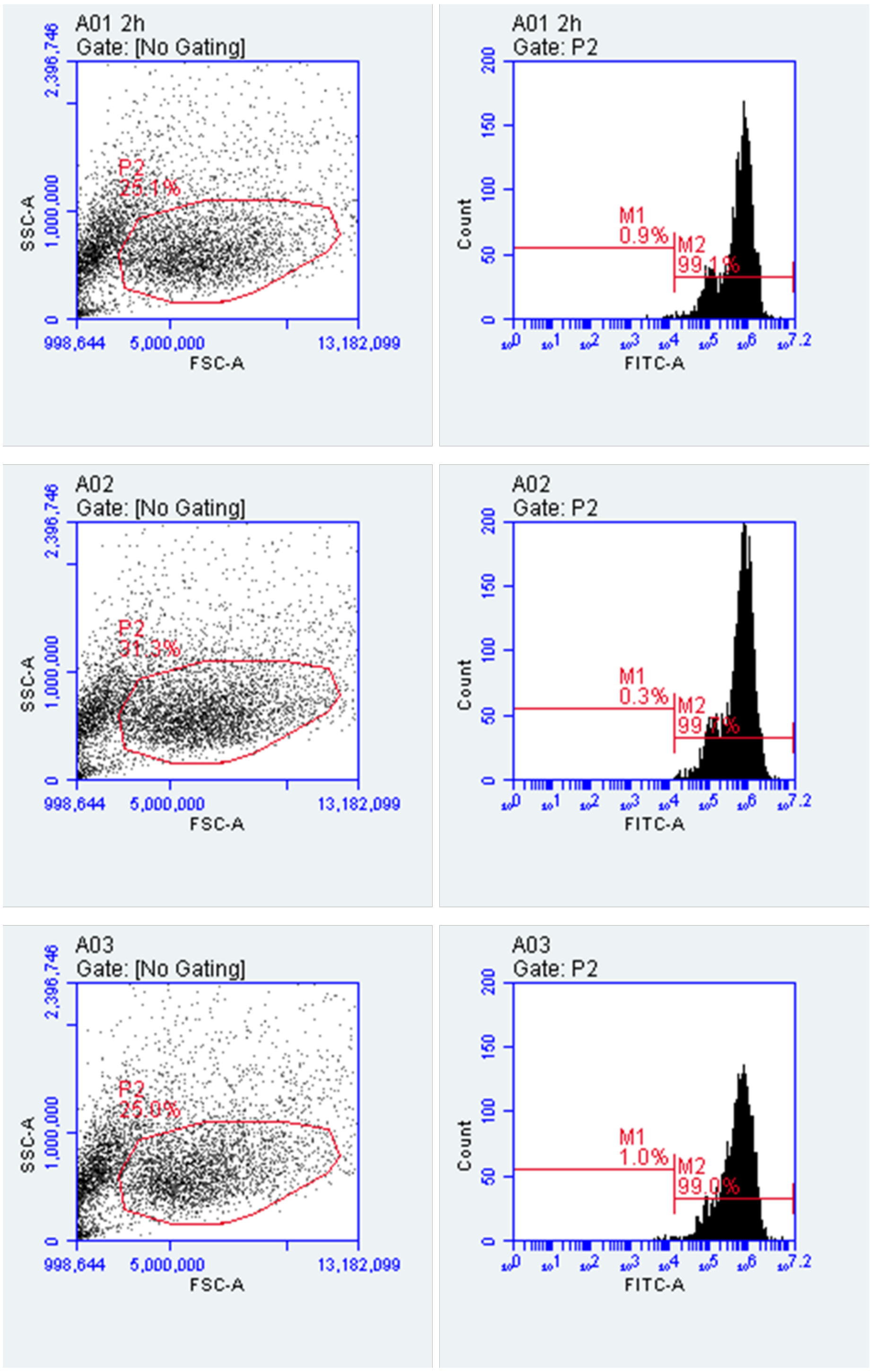
A01 2h
Gate: [No Gating]
P2
25.1%
SSC-A
FSC-A
998,644
5,000,000
13,182,099
2,396,746
1,000,000
A01 2h
Gate: P2
M1
0.9%
M2
99.1%
Count
FITC-A
A02
Gate: [No Gating]
P2
31.3%
A02
Gate: P2
M1
0.3%
M2
A03
Gate: [No Gating]
P2
25.0%
A03
Gate: P2
M1
1.0%
M2
99.0%

(b) **F-2 2 h**

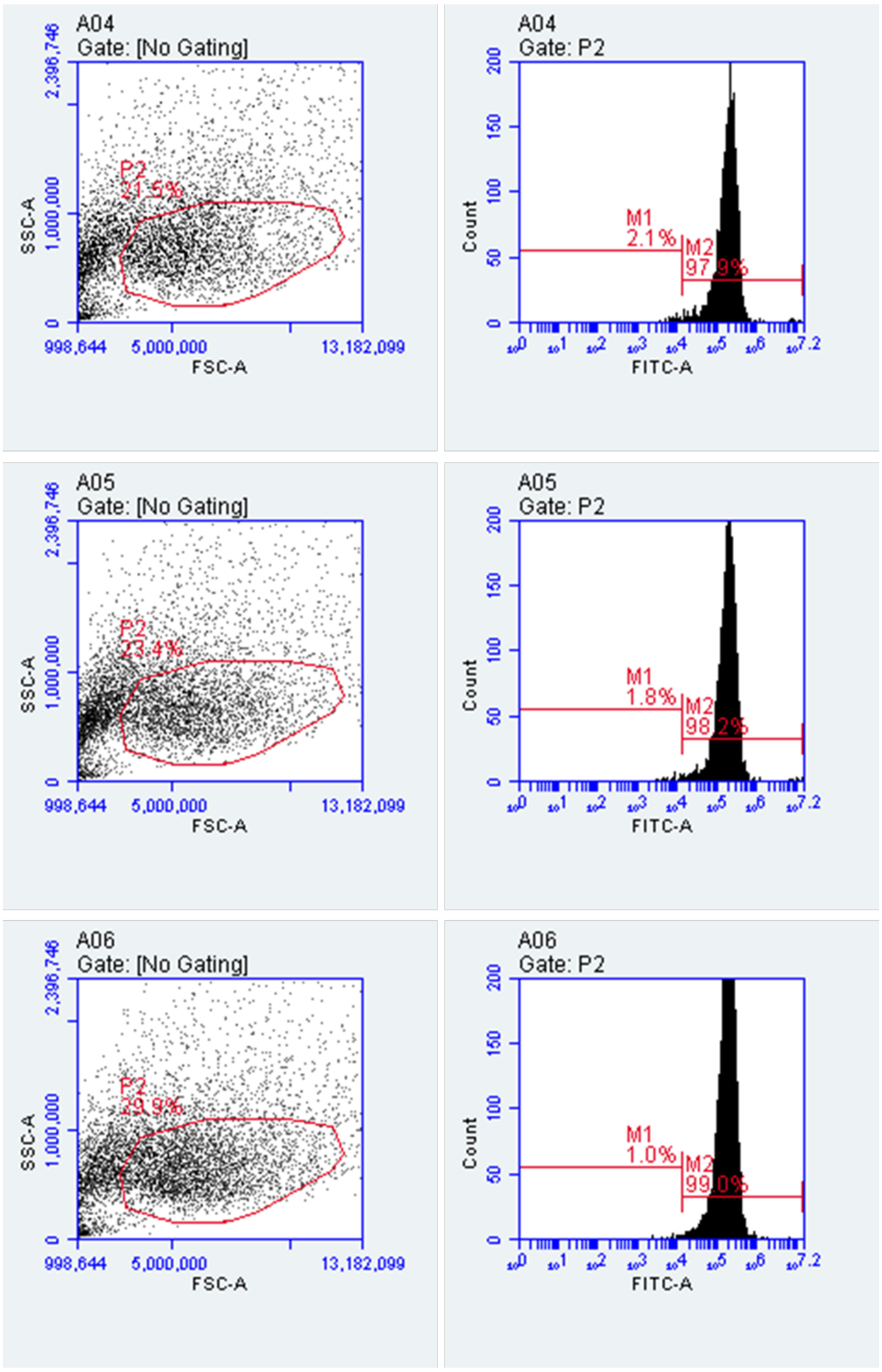
A04
Gate: [No Gating]
P2
21.5%
SSC-A
FSC-A
A04
Gate: P2
M1
2.1%
M2
97.9%
Count
FITC-A
A05
Gate: [No Gating]
P2
23.4%
A05
Gate: P2
M1
1.8%
M2
98.2%
A06
Gate: [No Gating]
P2
29.9%
A06
Gate: P2
M1
1.0%
M2
99.0%

(c) **F-3 2h**

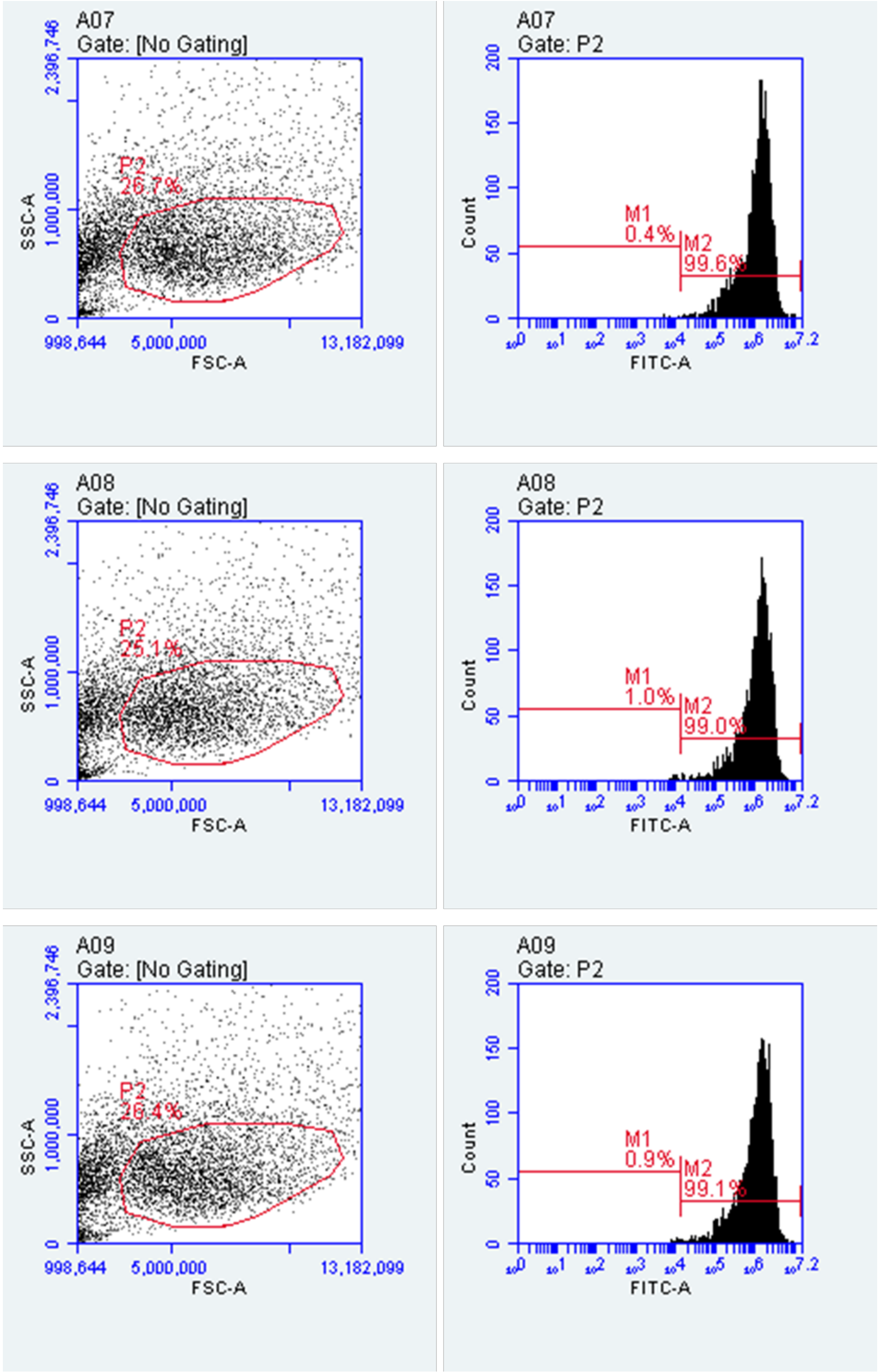
A07
Gate: [No Gating]
P2
26.7%
SSC-A
FSC-A
998,644
5,000,000
13,182,099
A07
Gate: P2
M1
0.4%
M2
99.6%
Count
FITC-A
A08
Gate: [No Gating]
P2
25.1%
A08
Gate: P2
M1
1.0%
M2
99.0%
A09
Gate: [No Gating]
P2
26.4%
A09
Gate: P2
M1
0.9%
M2
99.1%

(d) **F-4 2h**

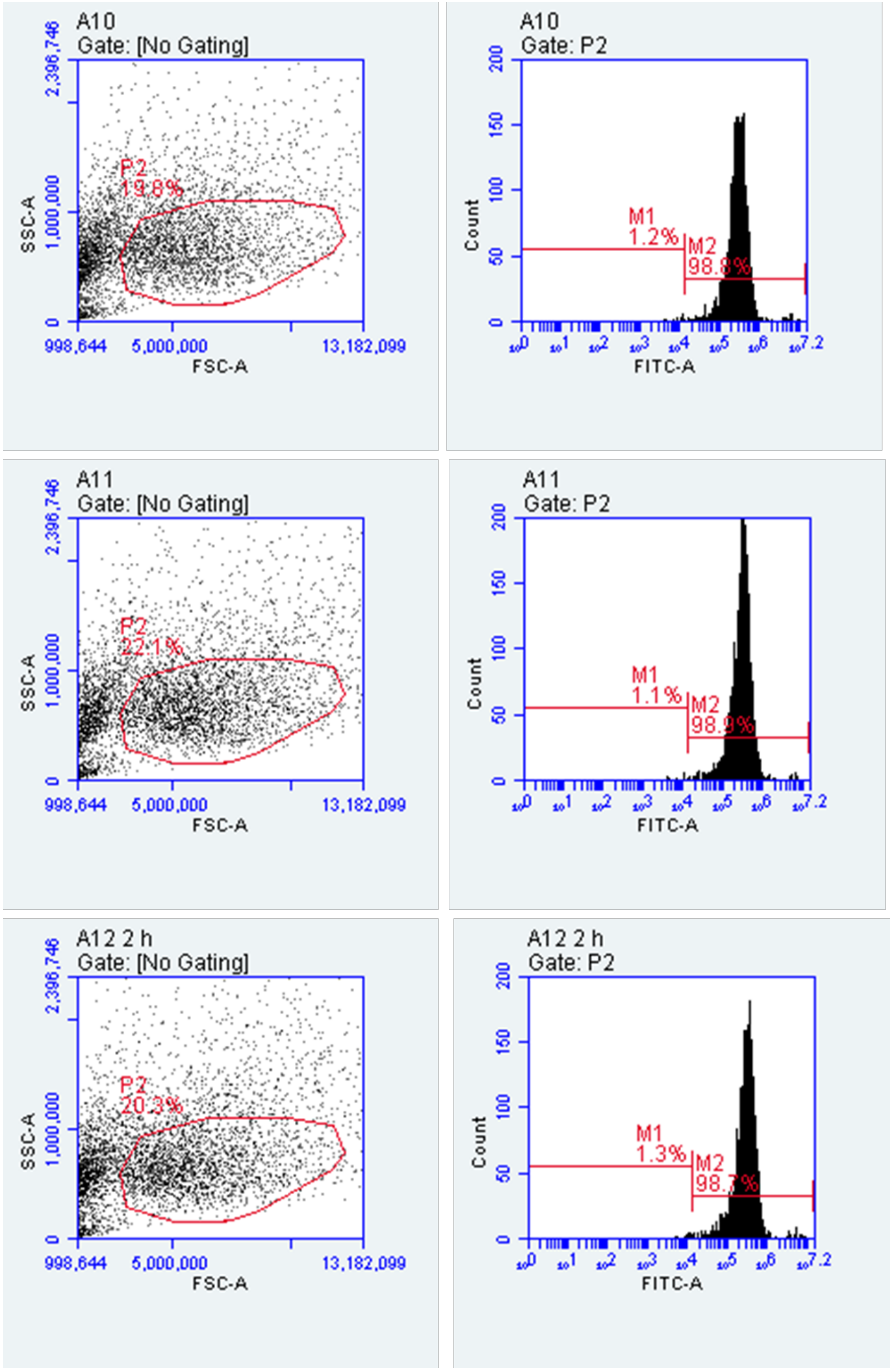

A10
Gate: [No Gating]
P2
19.8%
SSC-A
FSC-A
2,396,746
1,000,000
0
998,644
5,000,000
13,182,099
A10
Gate: P2
M1
1.2%
M2
98.8%
Count
FITC-A
A11
Gate: [No Gating]
P2
22.1%
A11
Gate: P2
M1
1.1%
M2
98.9%
A12 2 h
Gate: [No Gating]
P2
20.3%
A12 2 h
Gate: P2
M1
1.3%
M2
98.7%

(e) **F-1 48 h**

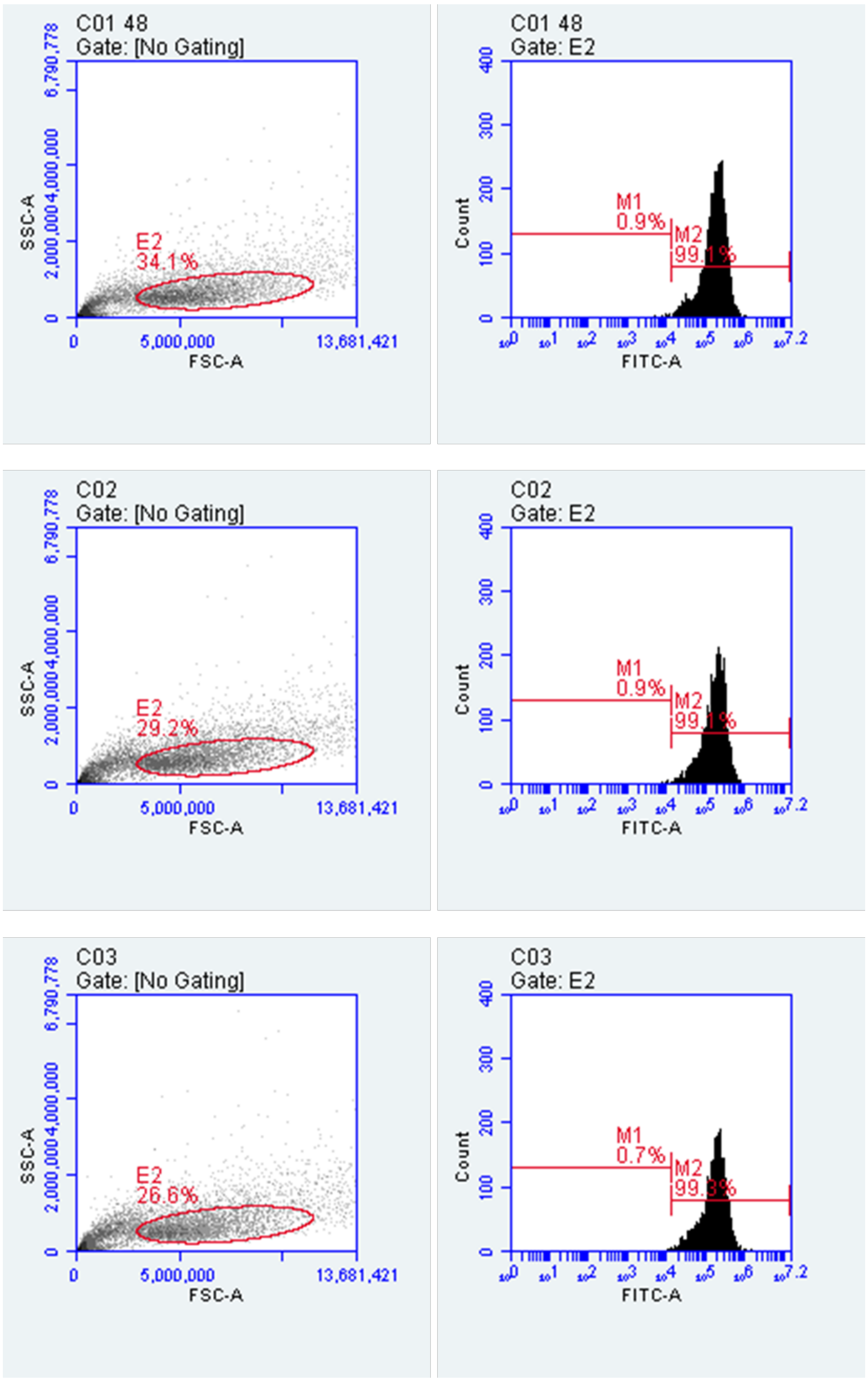
C01 48
Gate: [No Gating]
SSC-A
0
2,000,000
4,000,000
6,790,778
FSC-A
5,000,000
13,681,421
E2
34.1%
C01 48
Gate: E2
Count
M1
0.9%
M2
99.1%
FITC-A
C02
Gate: [No Gating]
E2
29.2%
C02
Gate: E2
M1
0.9%
M2
99.1%
C03
Gate: [No Gating]
E2
26.6%
C03
Gate: E2
M1
0.7%
M2
99.3%

(f) **F-2 48 h**

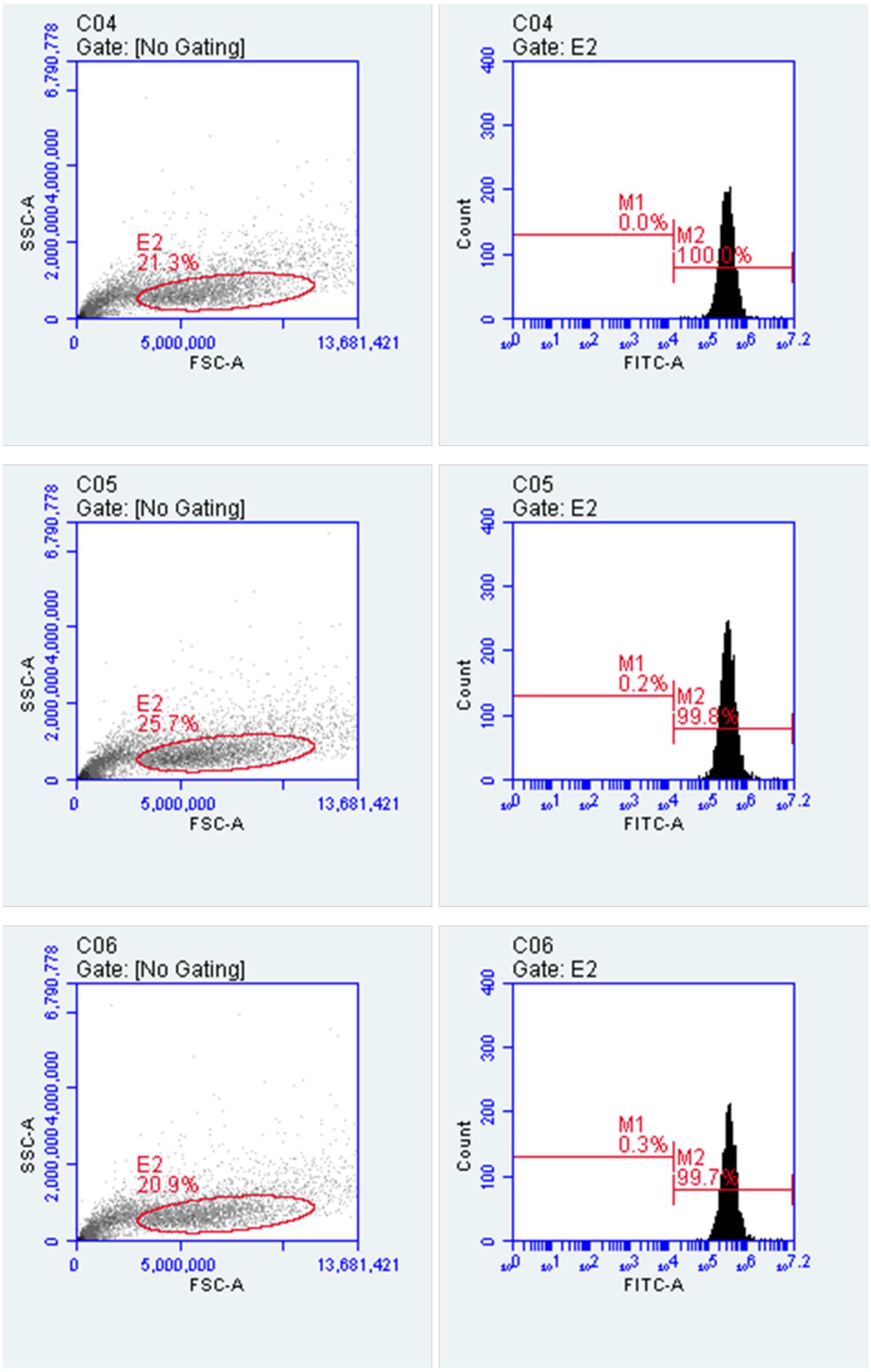
C04
Gate: [No Gating]
E2
21.3%
SSC-A
FSC-A
0
2,000,000
4,000,000
6,790,778
5,000,000
13,681,421
C04
Gate: E2
M1
0.0%
M2
100.0%
Count
FITC-A
0
100
200
300
400
C05
Gate: [No Gating]
E2
25.7%
C05
Gate: E2
M1
0.2%
M2
99.8%
C06
Gate: [No Gating]
E2
20.9%
C06
Gate: E2
M1
0.3%
M2
99.7%

(g) **F-3 48 h**

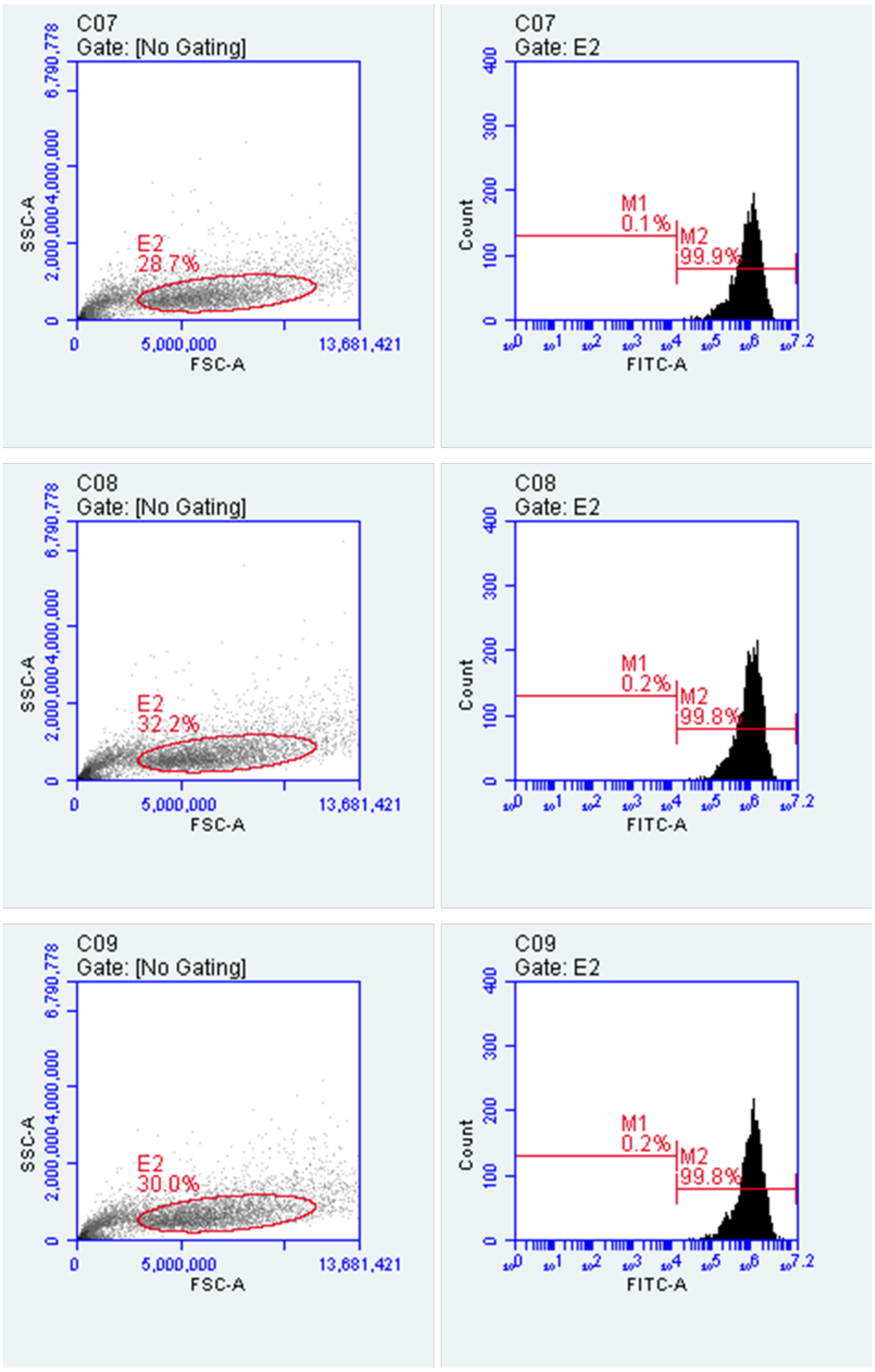
C07
Gate: [No Gating]
SSC-A
FSC-A
E2
28.7%
C07
Gate: E2
Count
FITC-A
M1
0.1%
M2
99.9%
C08
Gate: [No Gating]
SSC-A
FSC-A
E2
32.2%
C08
Gate: E2
Count
FITC-A
M1
0.2%
M2
99.8%
C09
Gate: [No Gating]
SSC-A
FSC-A
E2
30.0%
C09
Gate: E2
Count
FITC-A
M1
0.2%
M2
99.8%

(h) **F-4 48 h**

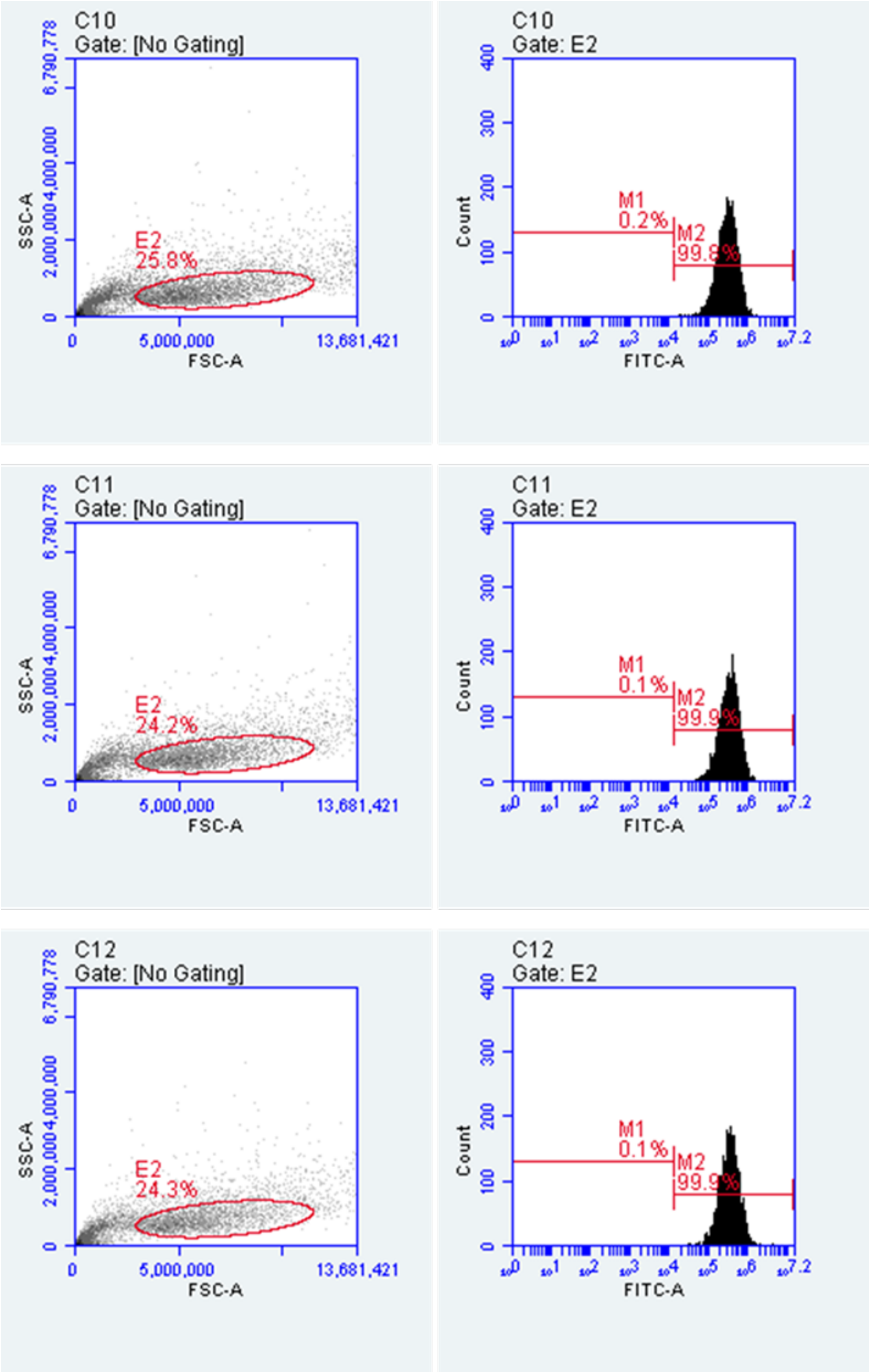

Figure S3. Raw data for scatter plots and histograms in flow cytometry in Figure 3. These data showed (a-d) 2 h incubation and (e-h) 48 h incubation. Each data set indicates n=3.

(a) **Blank without inhibitor**

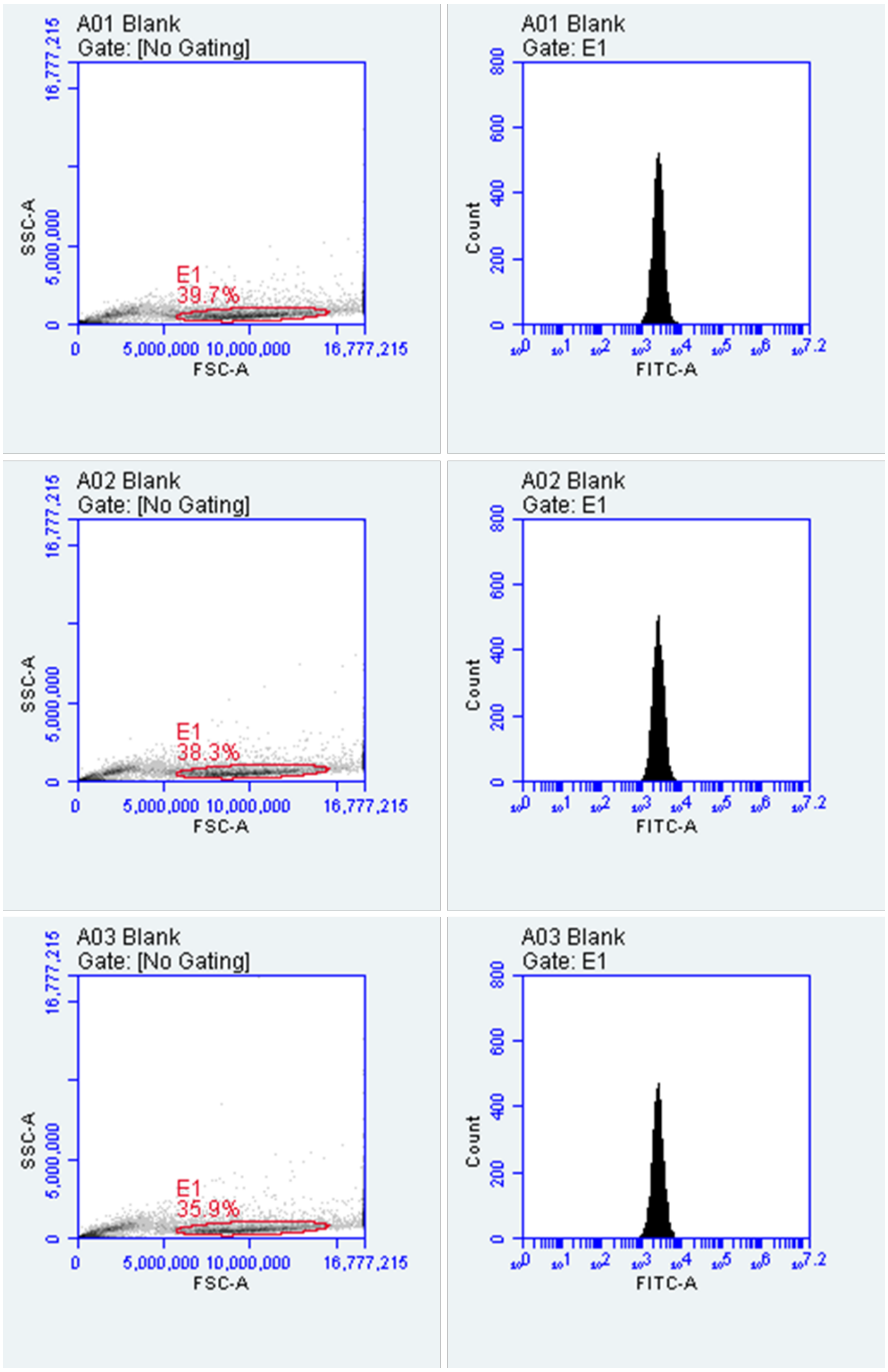

(b) **Blank amiloride**

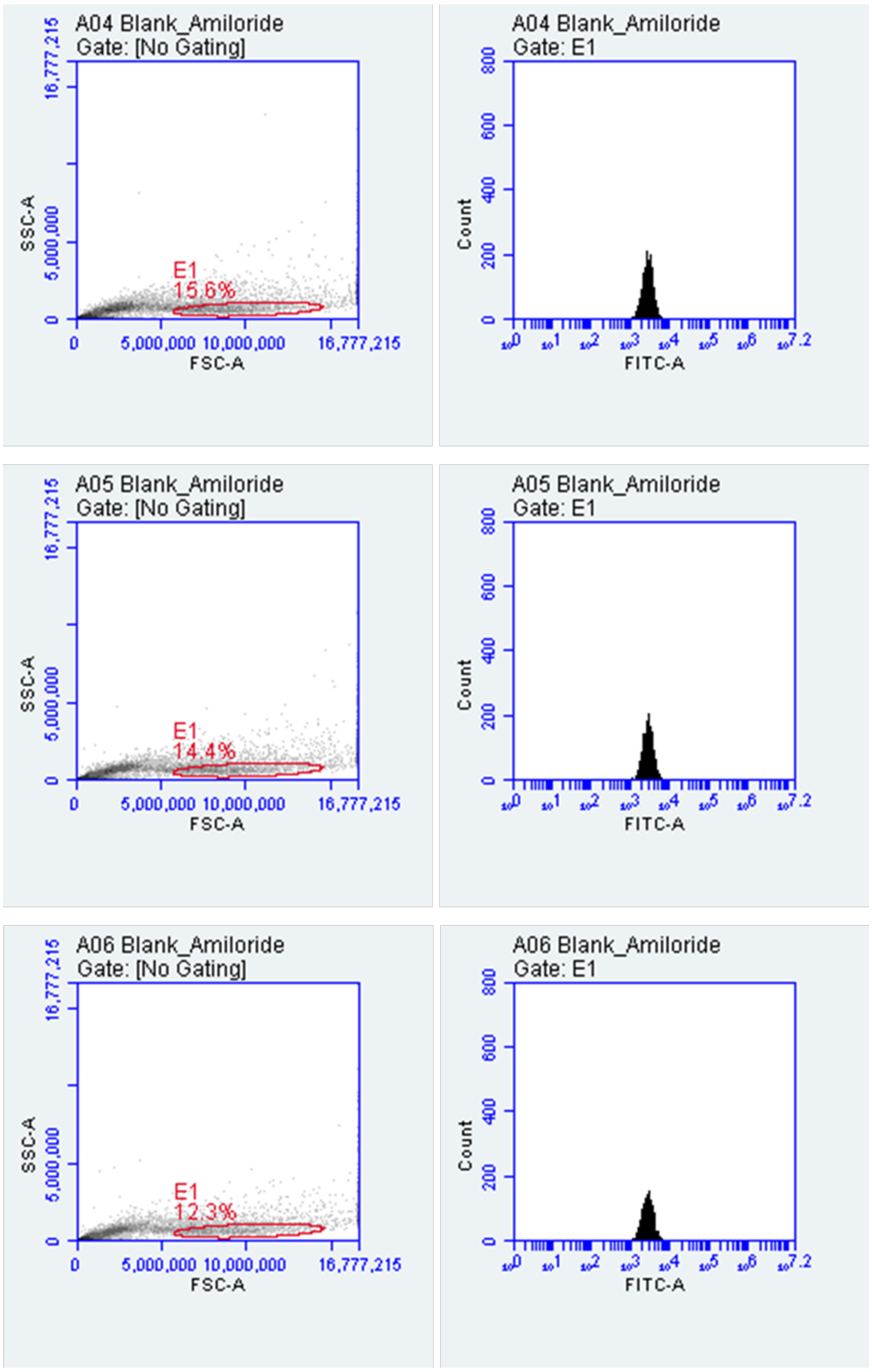

(c) **Blank nystatin**

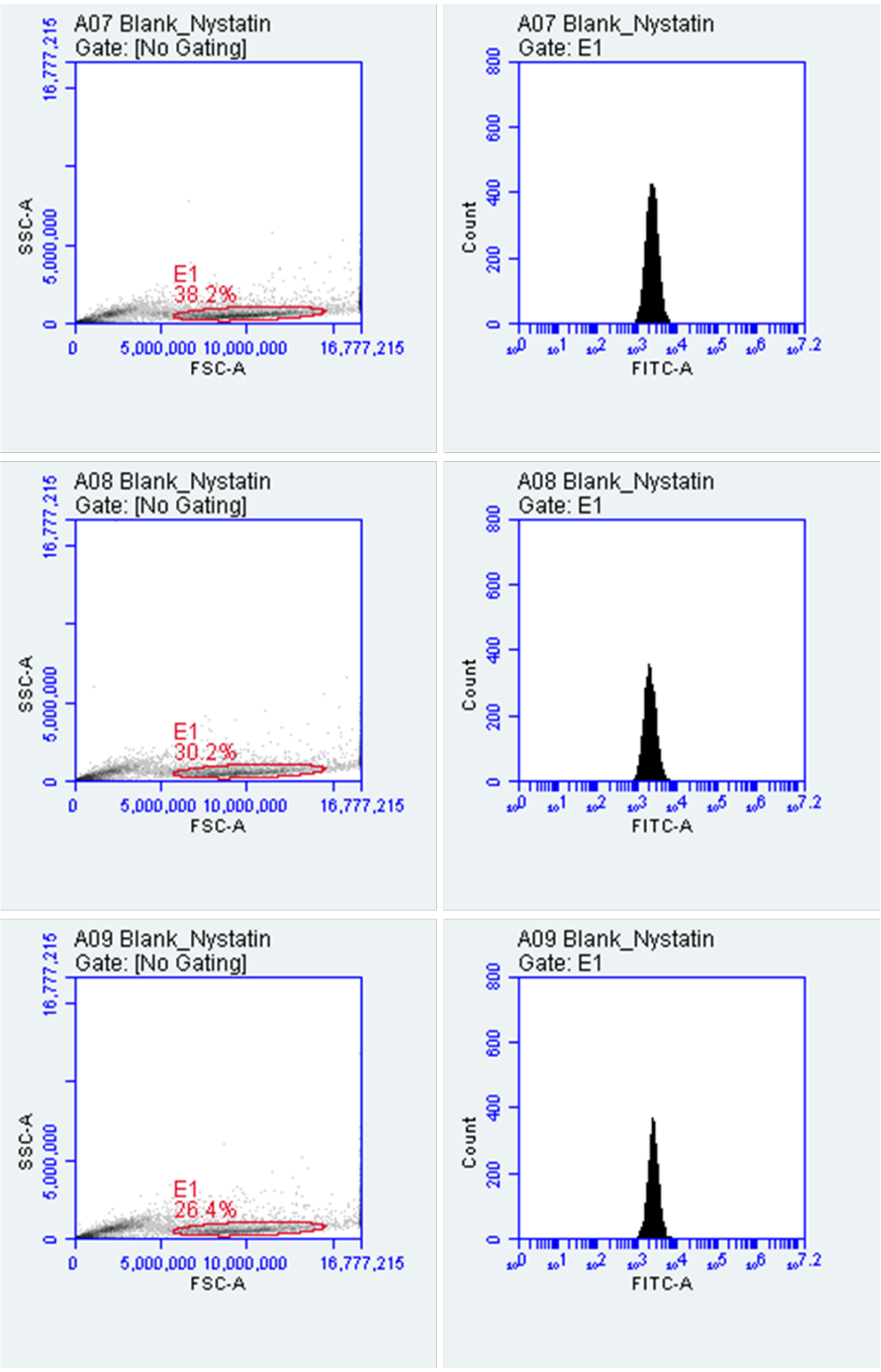

(d) **Blank sucrose**

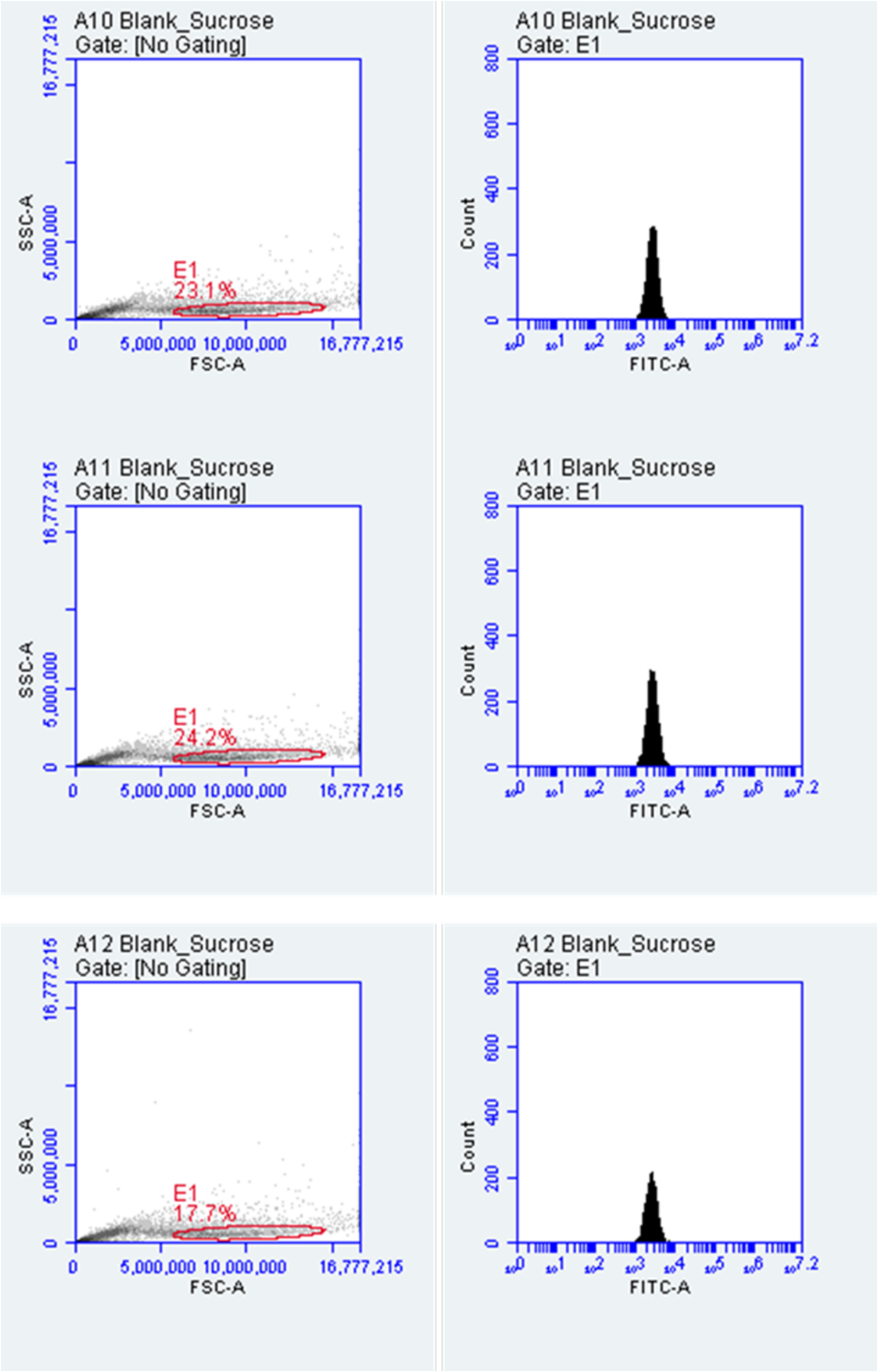

(e) **CF-R9 without inhibitor**

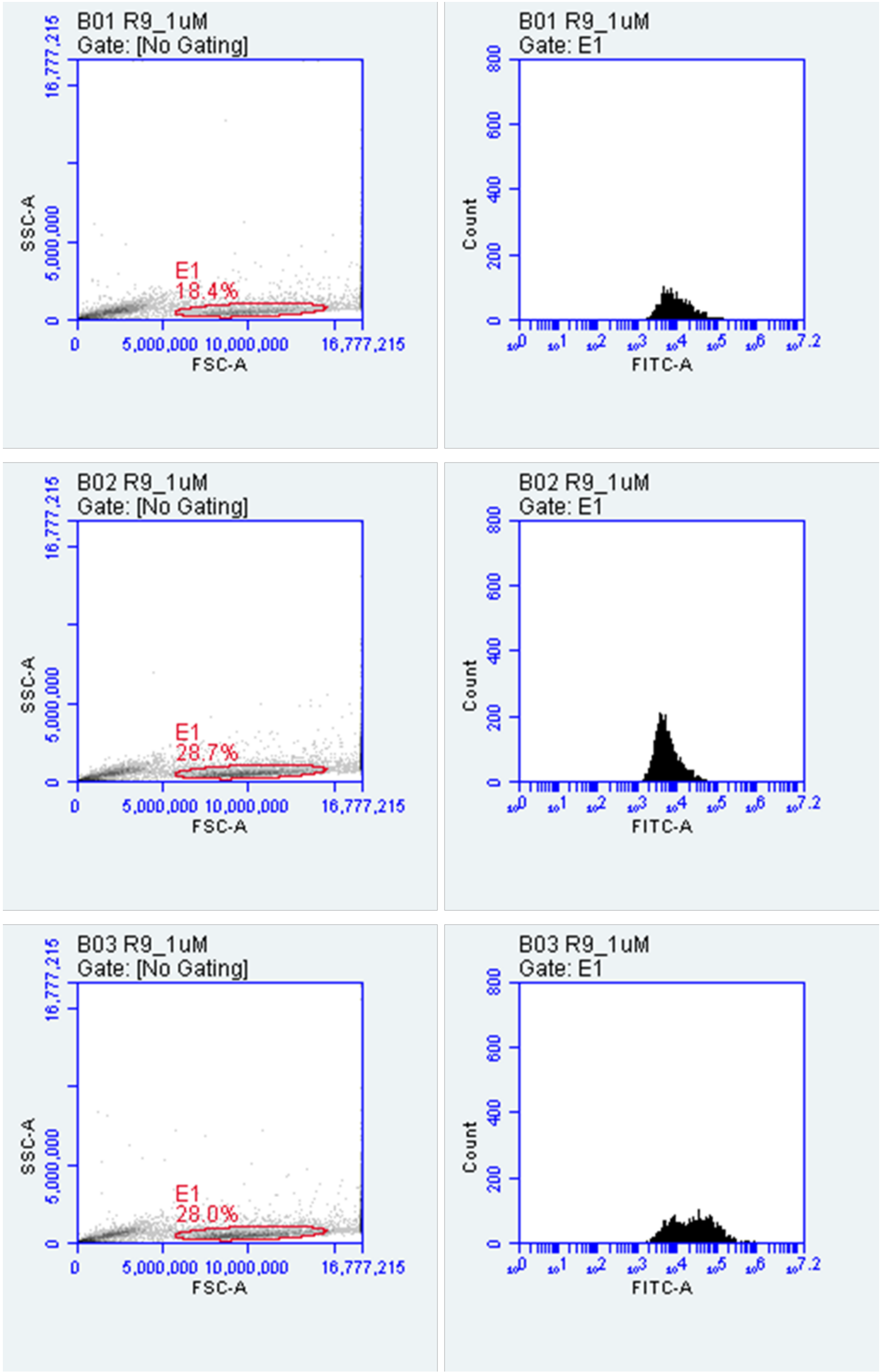

(f) **CF-R9 amiloride**

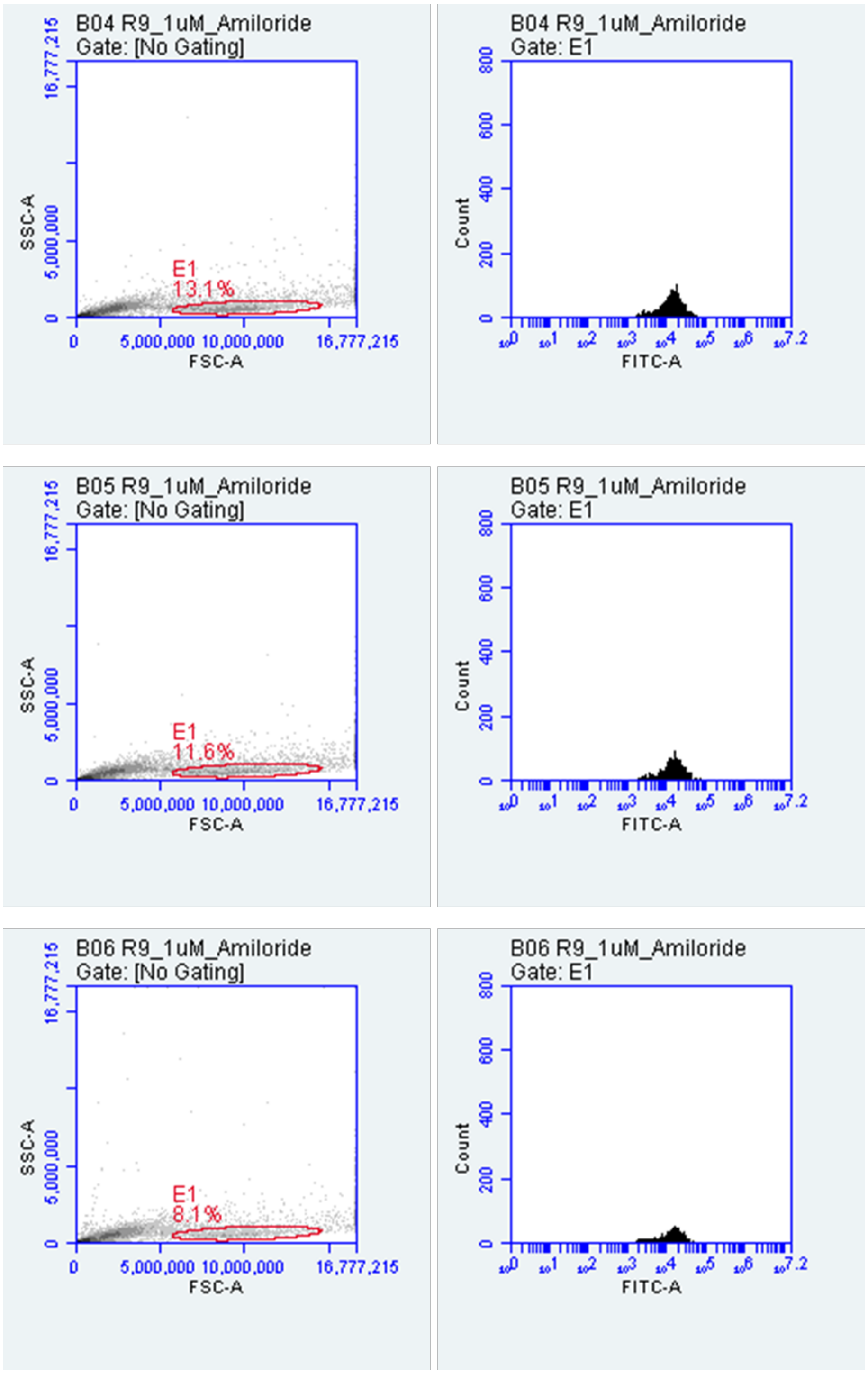

(g) **CF-R9 nystatin**

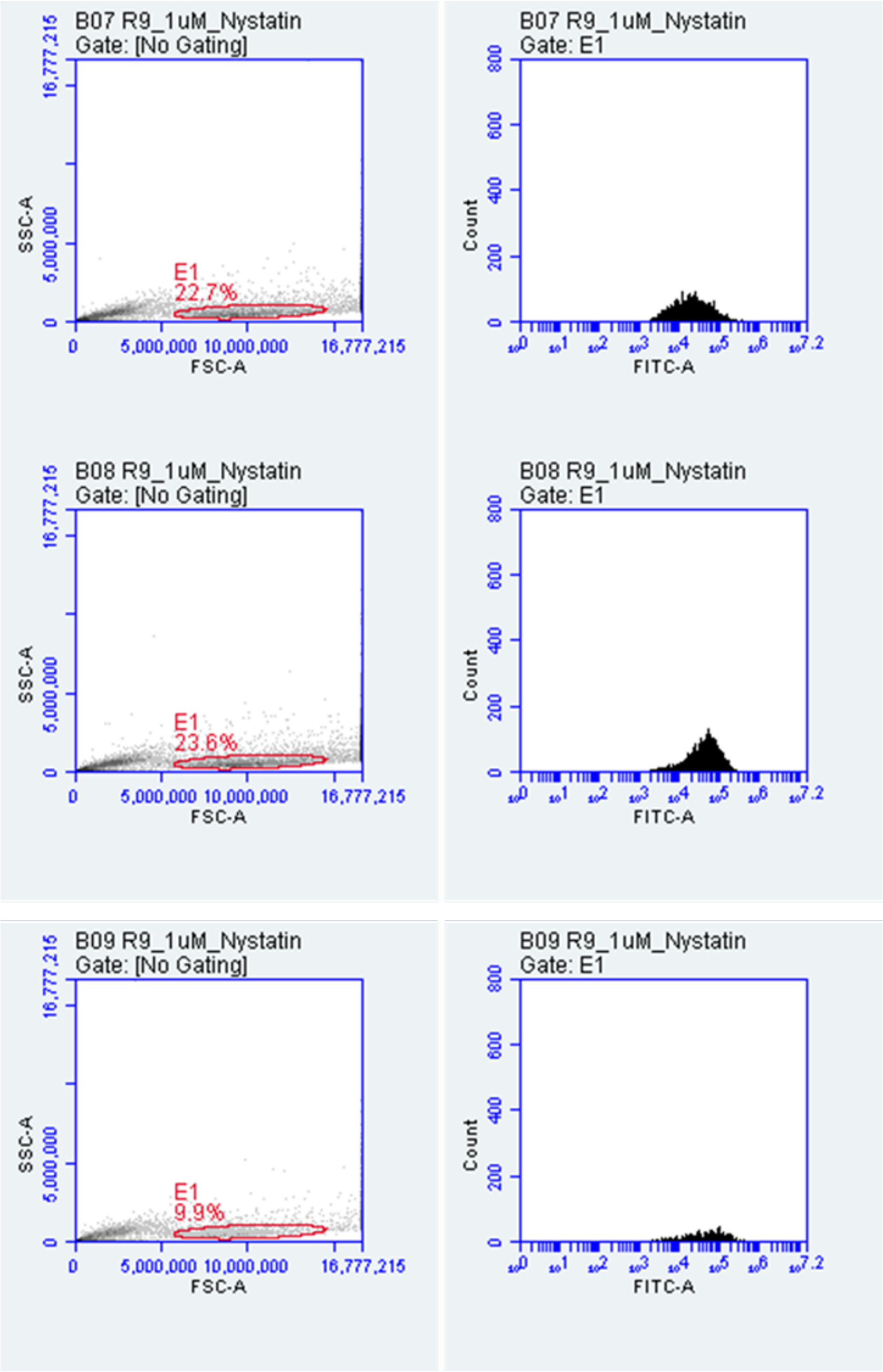

(h) **CF-R9 sucrose**

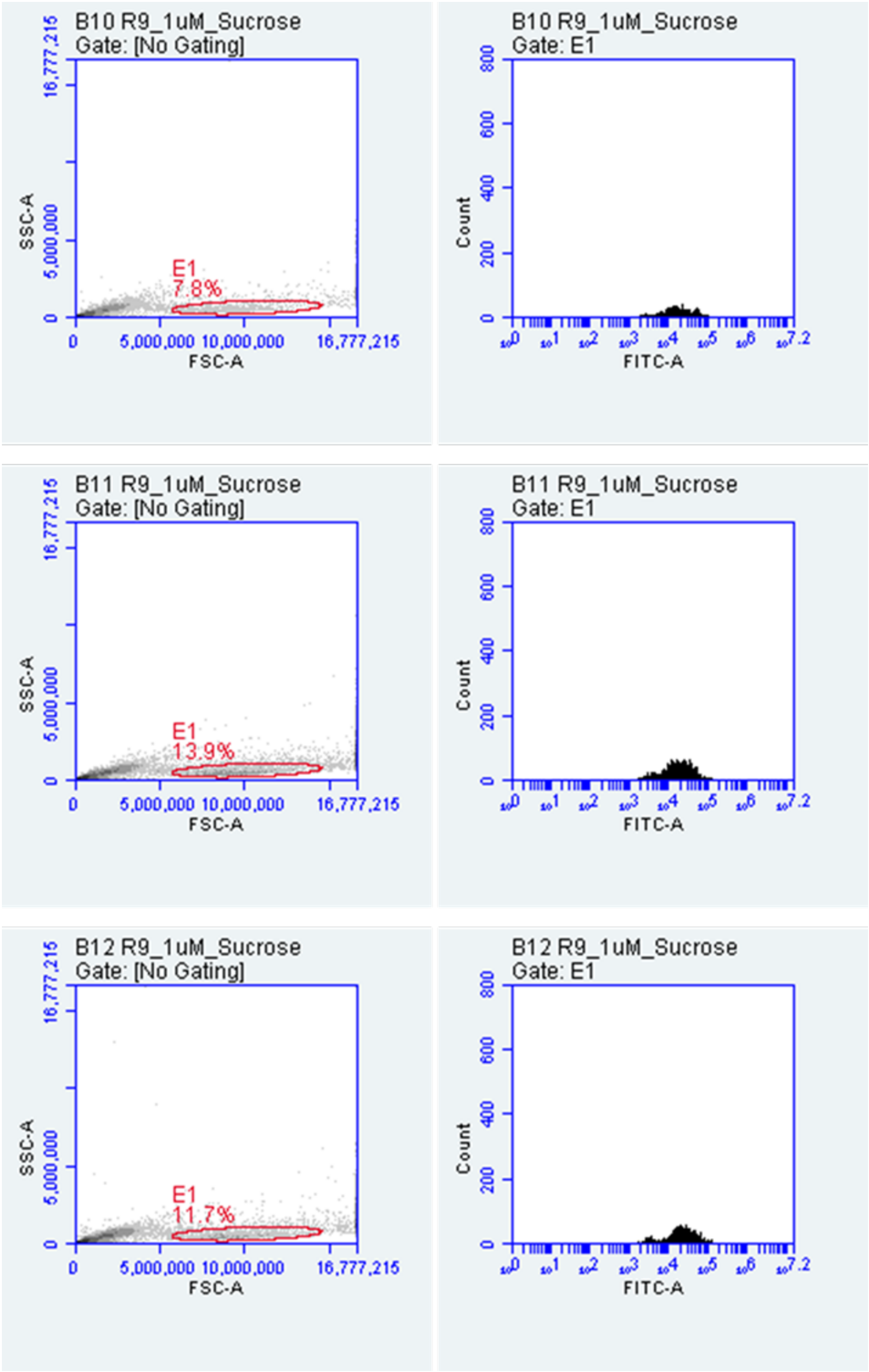

(i) **F-1 without inhibitor**

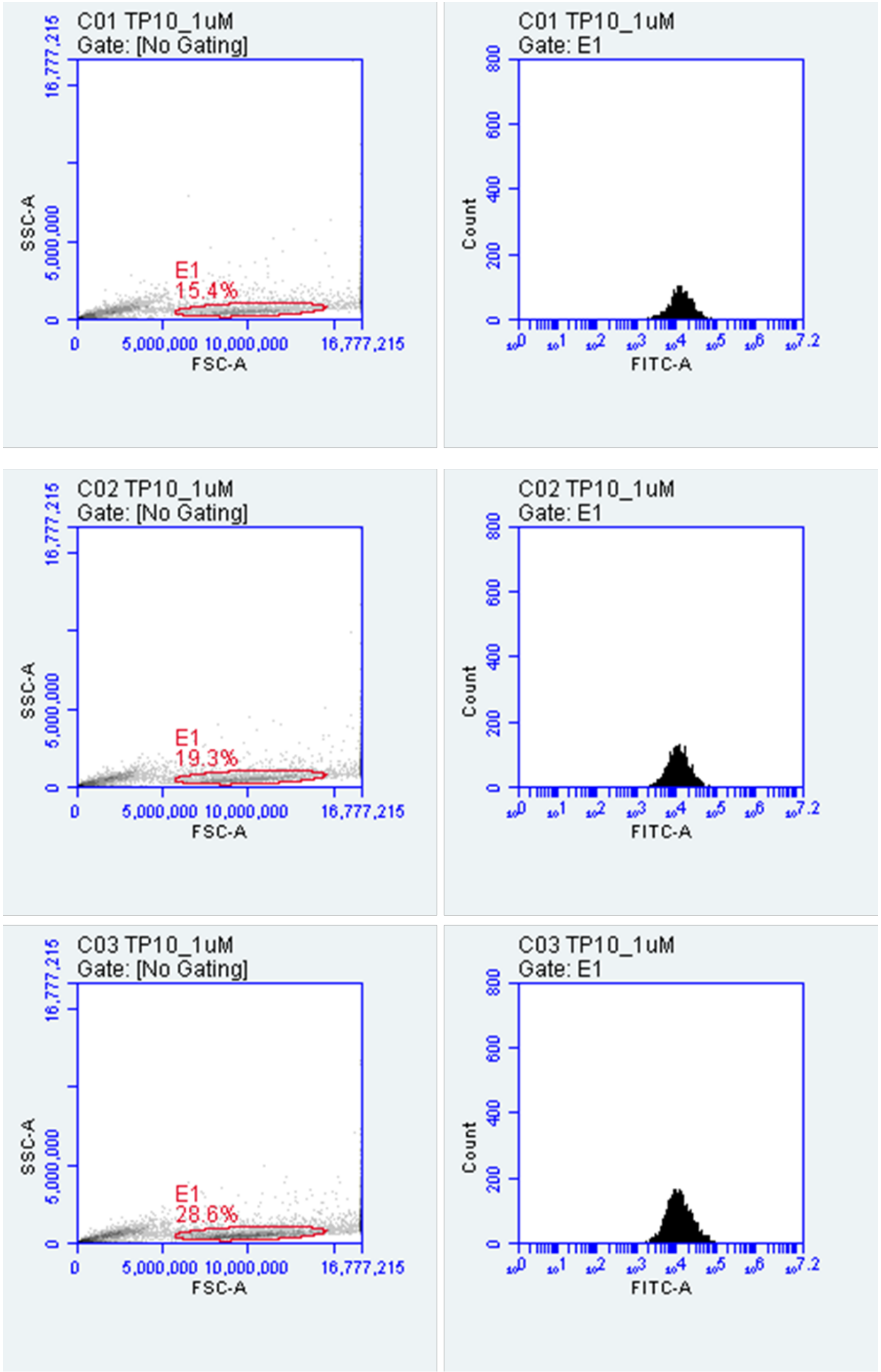

(j) **F-1 amiloride**

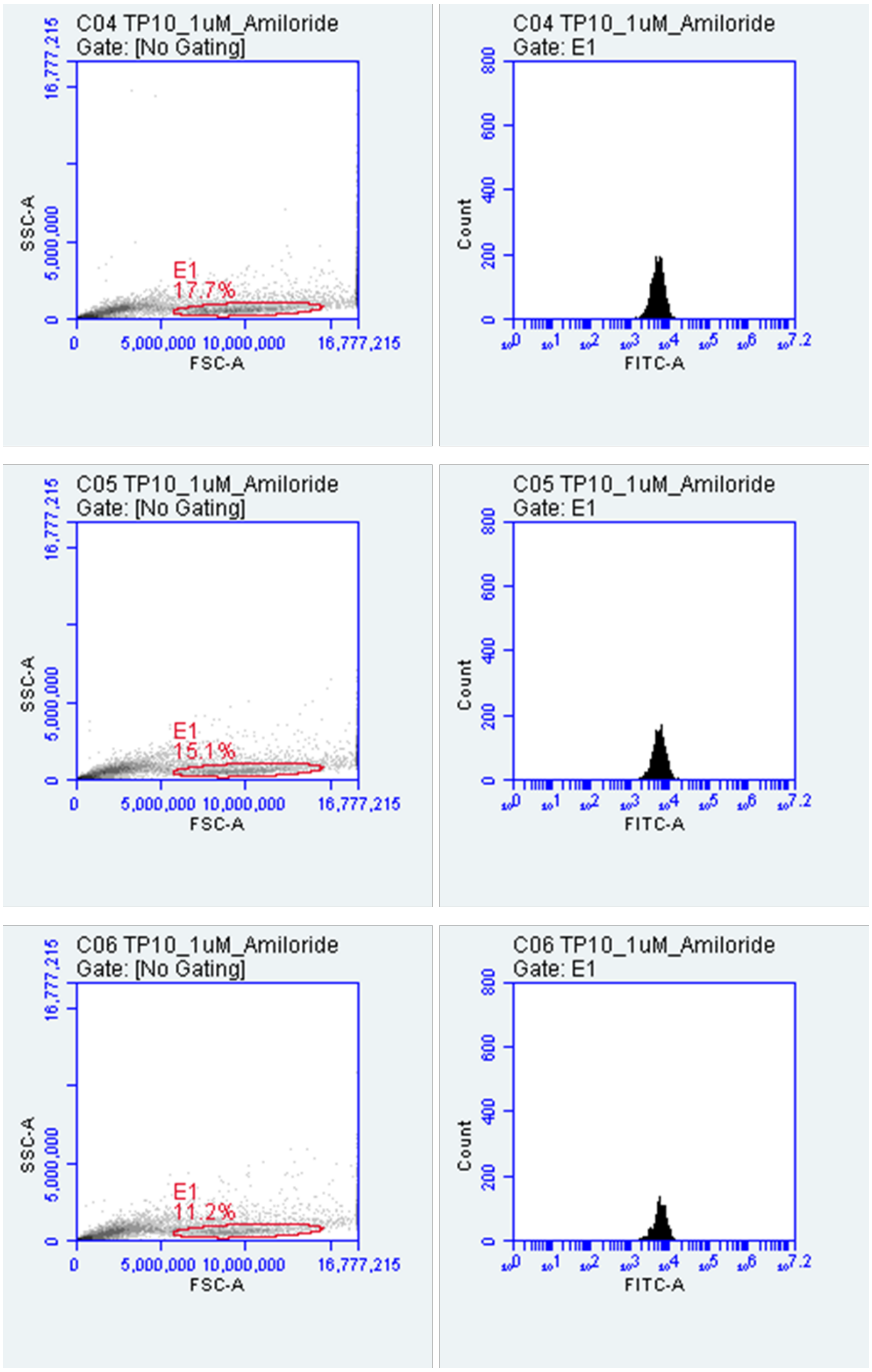

(k) **F--1 nystatin**

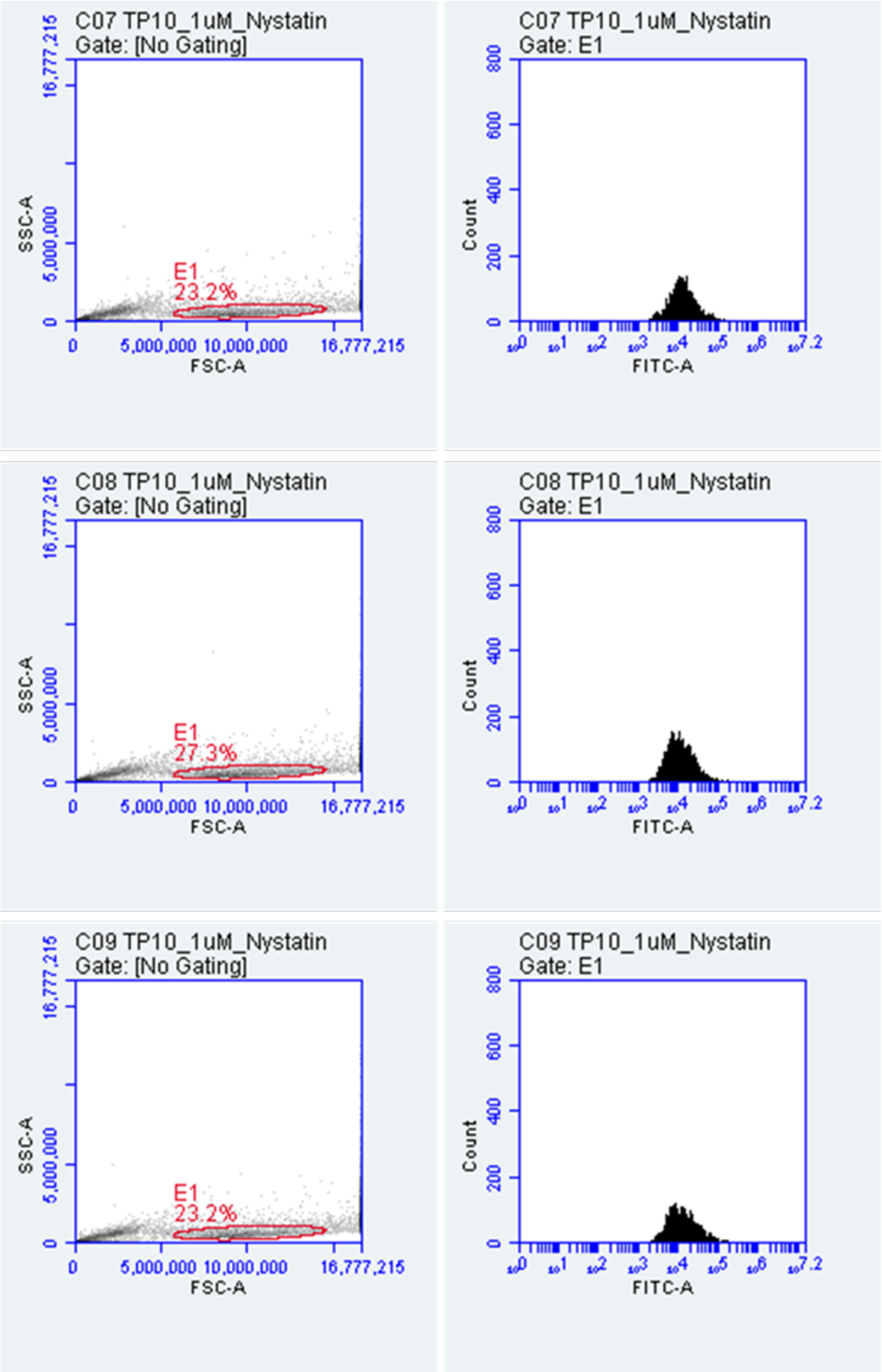
C07 TP10_1uM_Nystatin
Gate: [No Gating]
SSC-A
FSC-A
E1
23.2%
0
5,000,000
10,000,000
16,777,215
C07 TP10_1uM_Nystatin
Gate: E1
Count
FITC-A
0
200
400
600
800
C08 TP10_1uM_Nystatin
Gate: [No Gating]
E1
27.3%
C08 TP10_1uM_Nystatin
Gate: E1
C09 TP10_1uM_Nystatin
Gate: [No Gating]
E1
23.2%
C09 TP10_1uM_Nystatin
Gate: E1

(l) **F-1 sucrose**

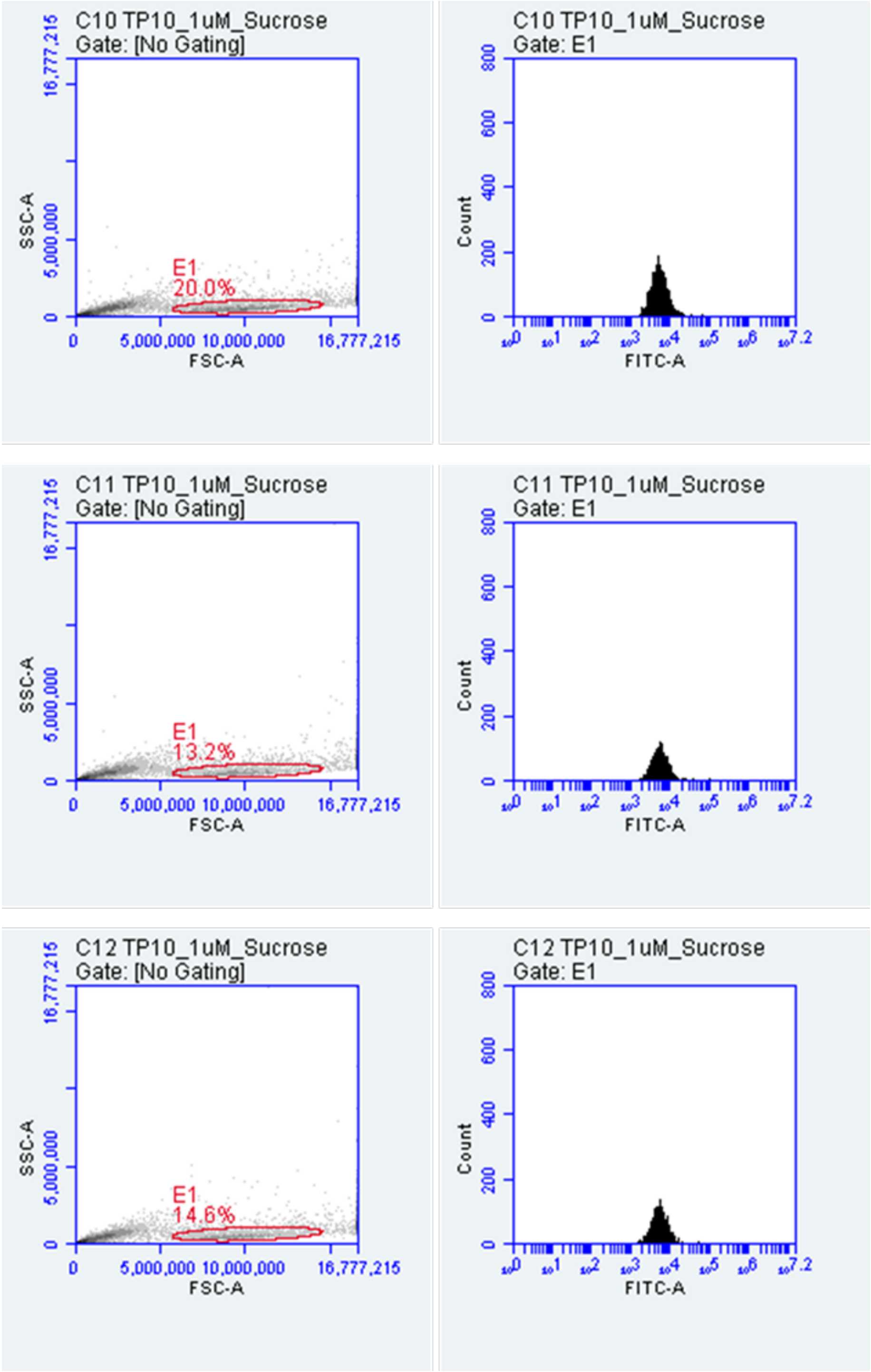
C10 TP10_1uM_Sucrose
Gate: [No Gating]
E1
20.0%
SSC-A
FSC-A
C10 TP10_1uM_Sucrose
Gate: E1
Count
FITC-A
C11 TP10_1uM_Sucrose
Gate: [No Gating]
E1
13.2%
SSC-A
FSC-A
C11 TP10_1uM_Sucrose
Gate: E1
Count
FITC-A
C12 TP10_1uM_Sucrose
Gate: [No Gating]
E1
14.6%
SSC-A
FSC-A
C12 TP10_1uM_Sucrose
Gate: E1
Count
FITC-A

(m) **F-3 without inhibitor**

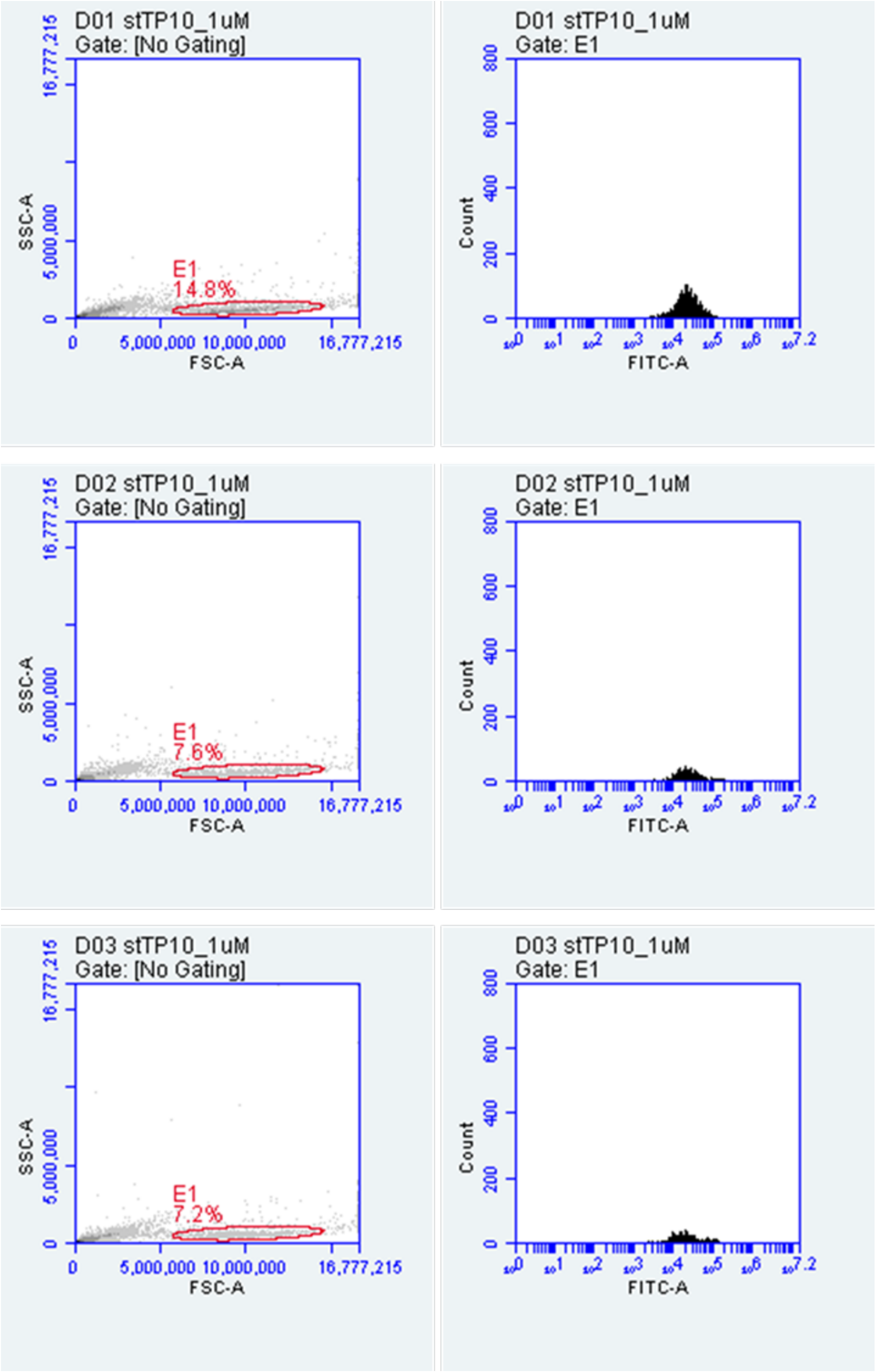

(n) **F-3 + amiloride**

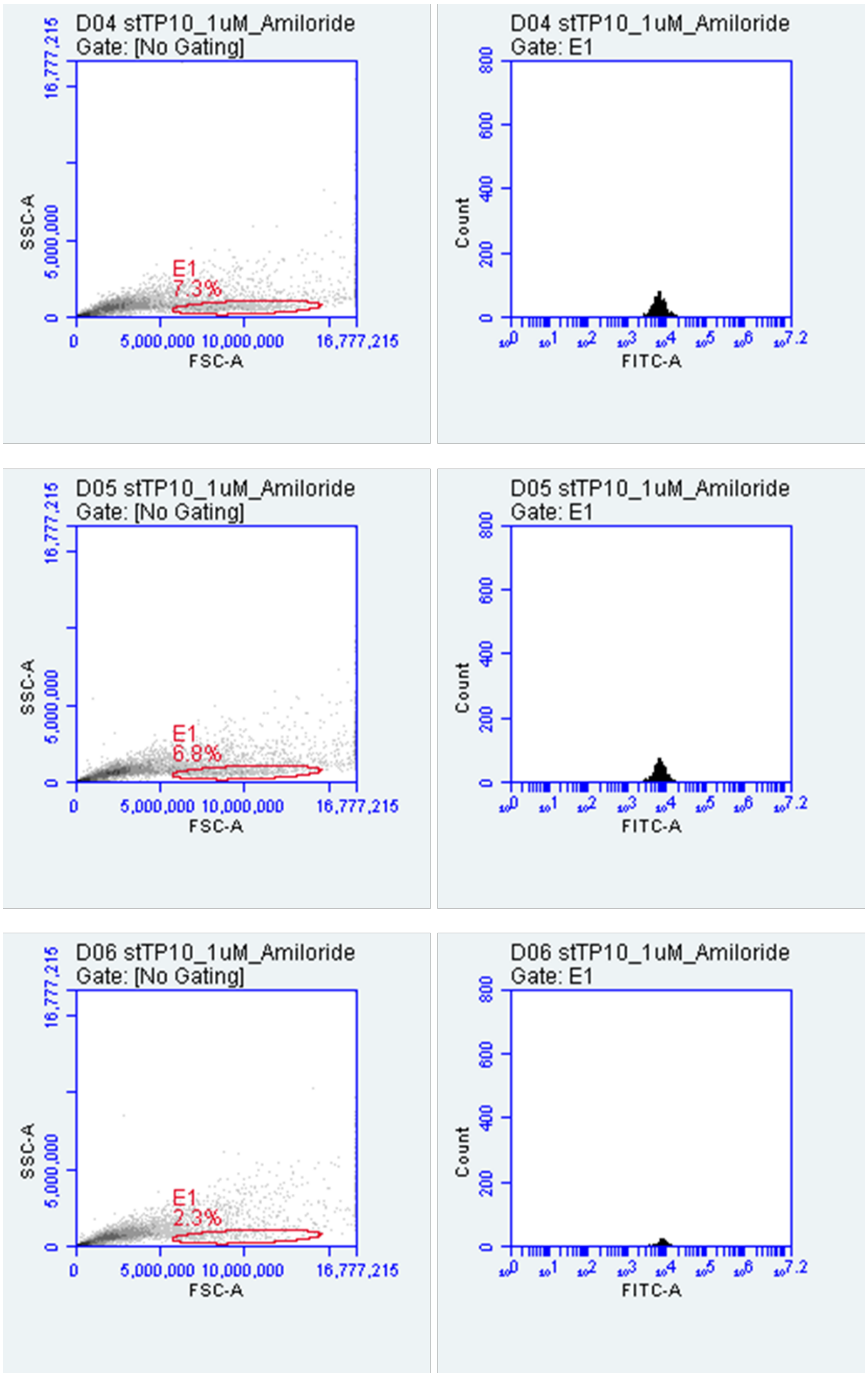

(o) **F-3 nystatin**

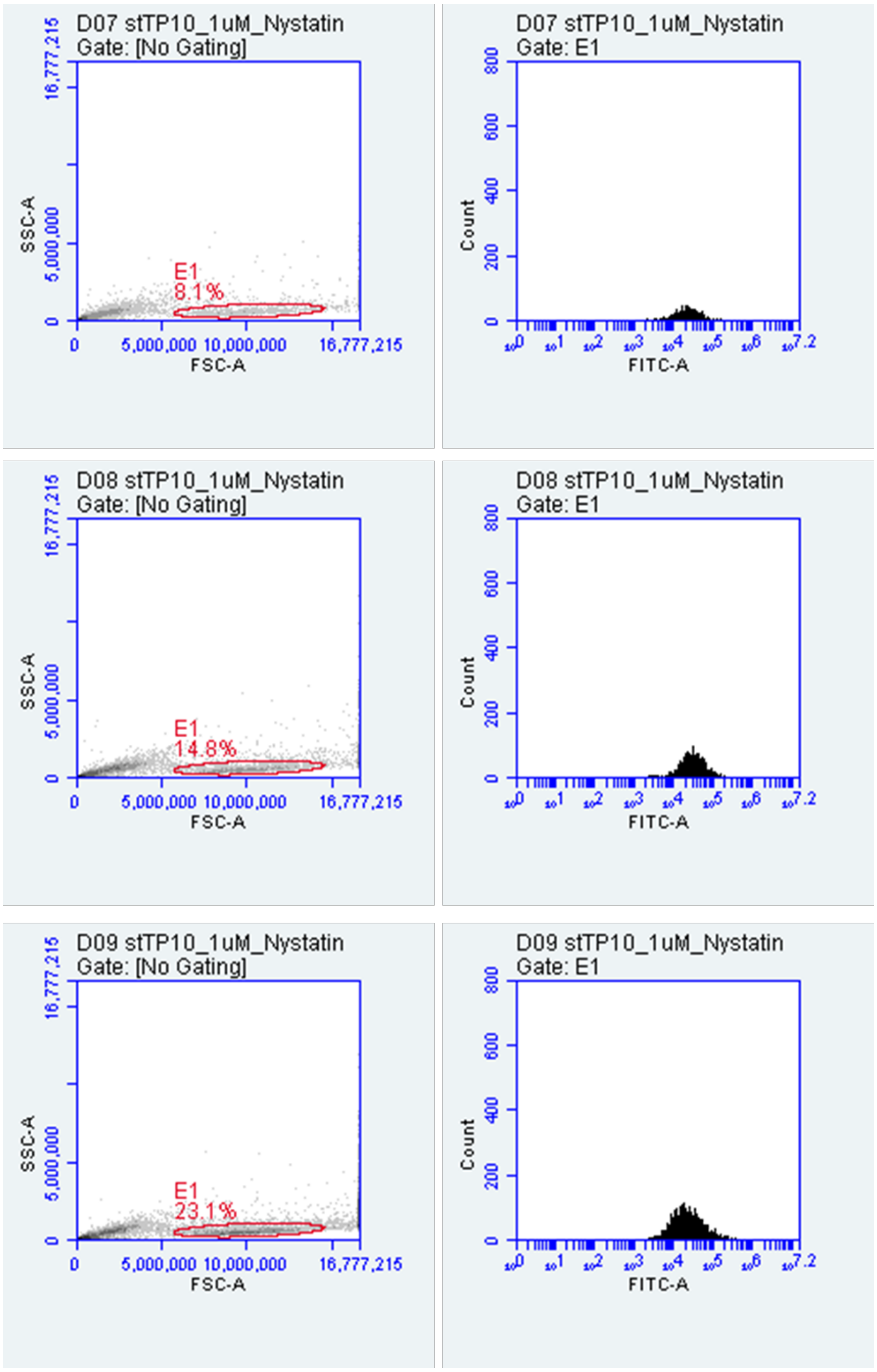

(p) **F-3 sucrose**

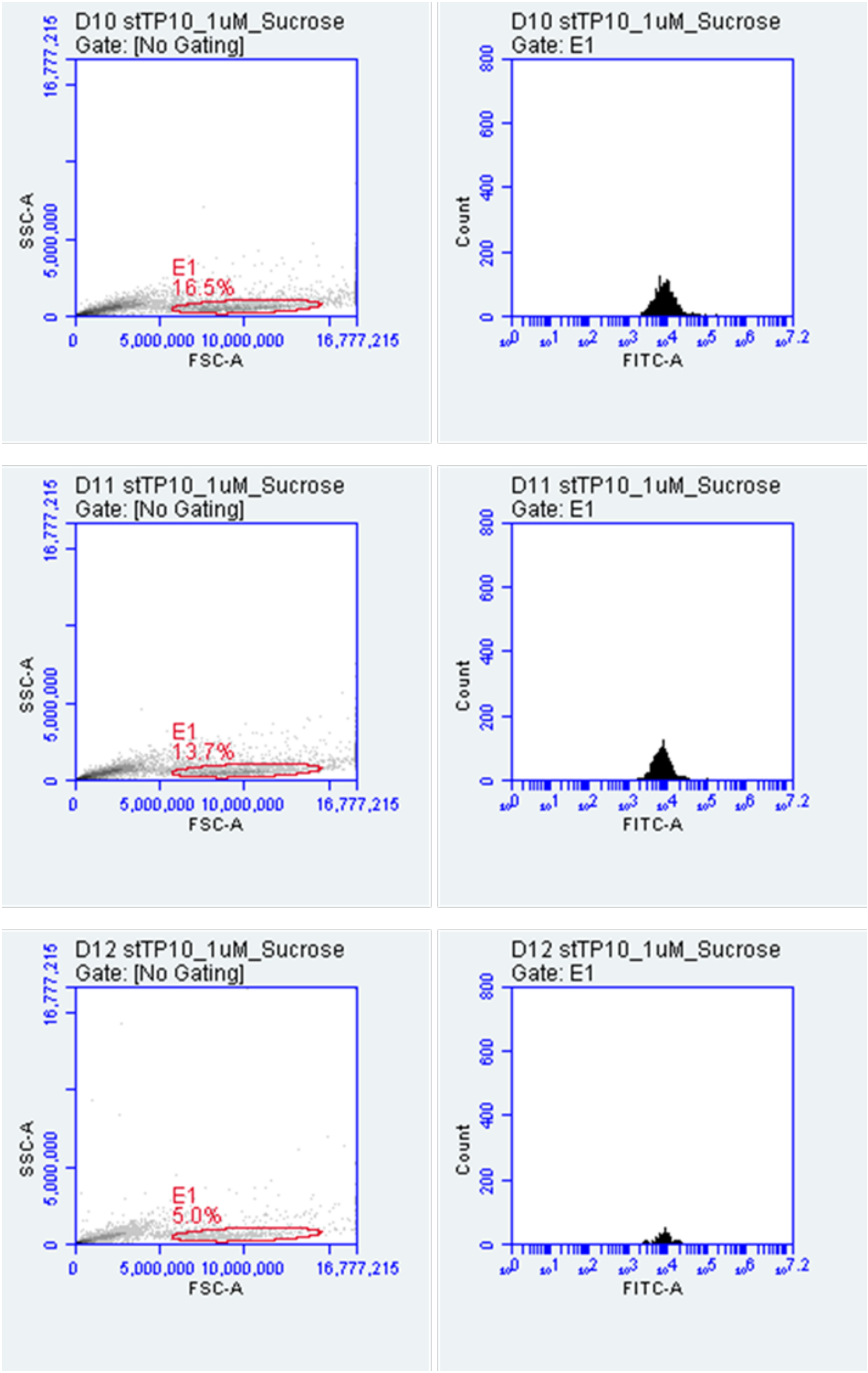

Figure S4. Raw data for scatter plots and histograms in flow cytometry in Figure 4a. These data showed treatment of each endocytosis inhibitor (a-d) and, the peptide and respective endocytosis inhibitor co-additive conditions (e-p) . Each data set indicates n=3.

(a) **CF-R9 4 ° C**

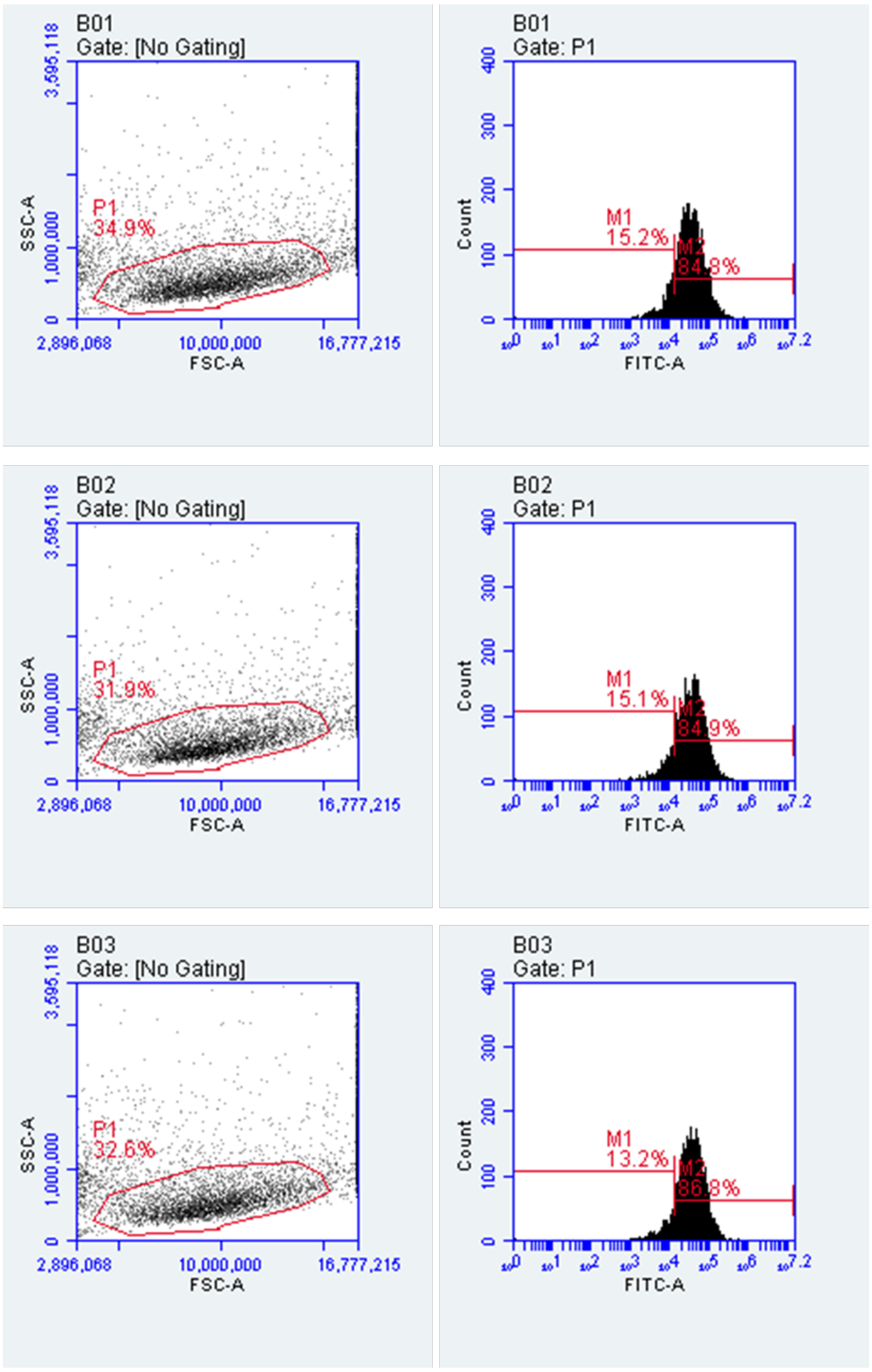
B01
Gate: [No Gating]
P1
34.9%
SSC-A
FSC-A
B01
Gate: P1
M1
15.2%
M2
84.8%
Count
FITC-A
B02
Gate: [No Gating]
P1
31.9%
B02
Gate: P1
M1
15.1%
M2
84.9%
B03
Gate: [No Gating]
P1
32.6%
B03
Gate: P1
M1
13.2%
M2
86.8%

(b) **CF-R9 37 °C**

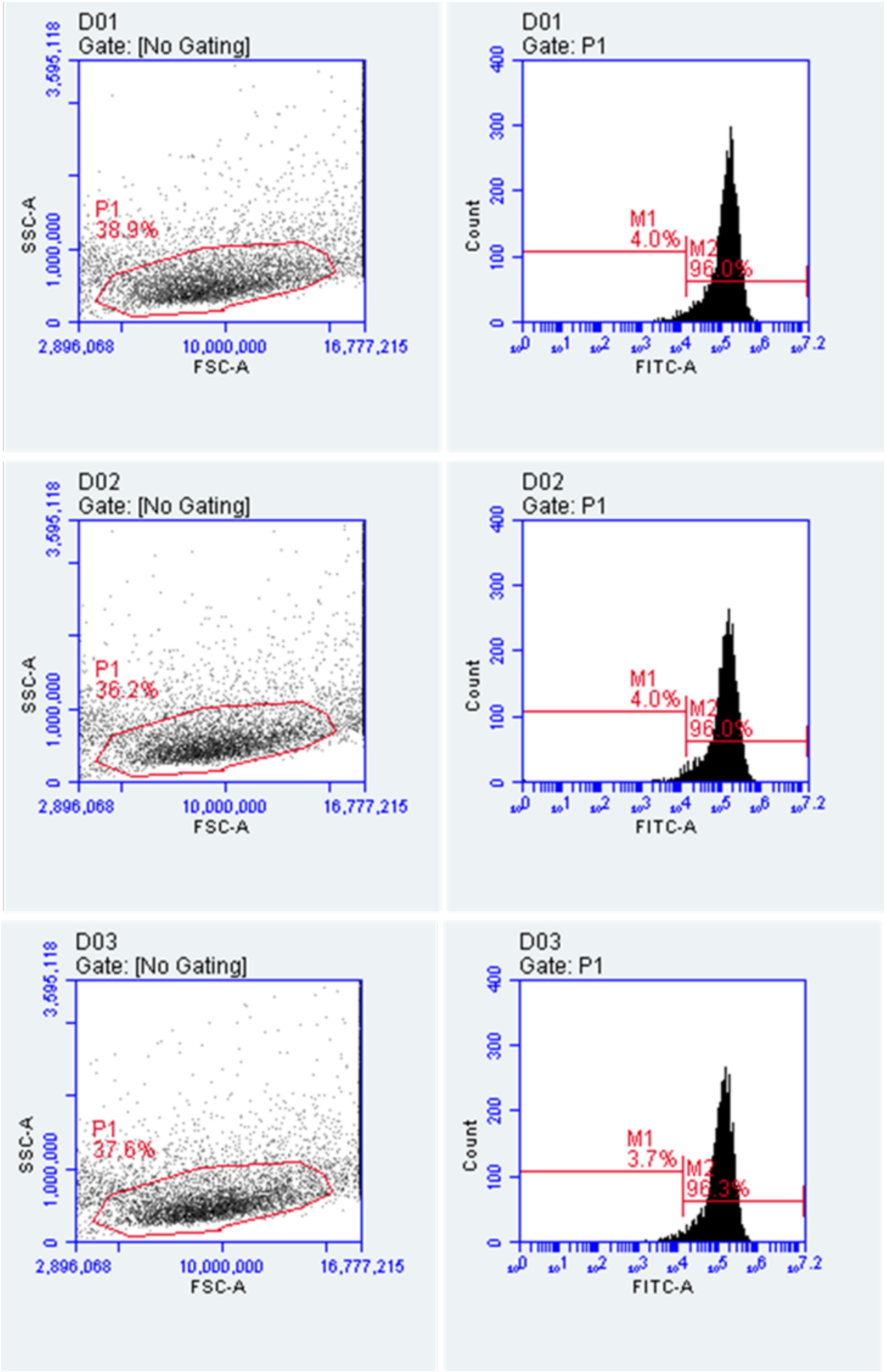
D01
Gate: [No Gating]
P1
38.9%
SSC-A
FSC-A
D01
Gate: P1
M1
4.0%
M2
96.0%
Count
FITC-A
D02
Gate: [No Gating]
P1
36.2%
SSC-A
FSC-A
D02
Gate: P1
M1
4.0%
M2
96.0%
Count
FITC-A
D03
Gate: [No Gating]
P1
37.6%
SSC-A
FSC-A
D03
Gate: P1
M1
3.7%
M2
96.3%
Count
FITC-A

(c) **F-1 4 °C**

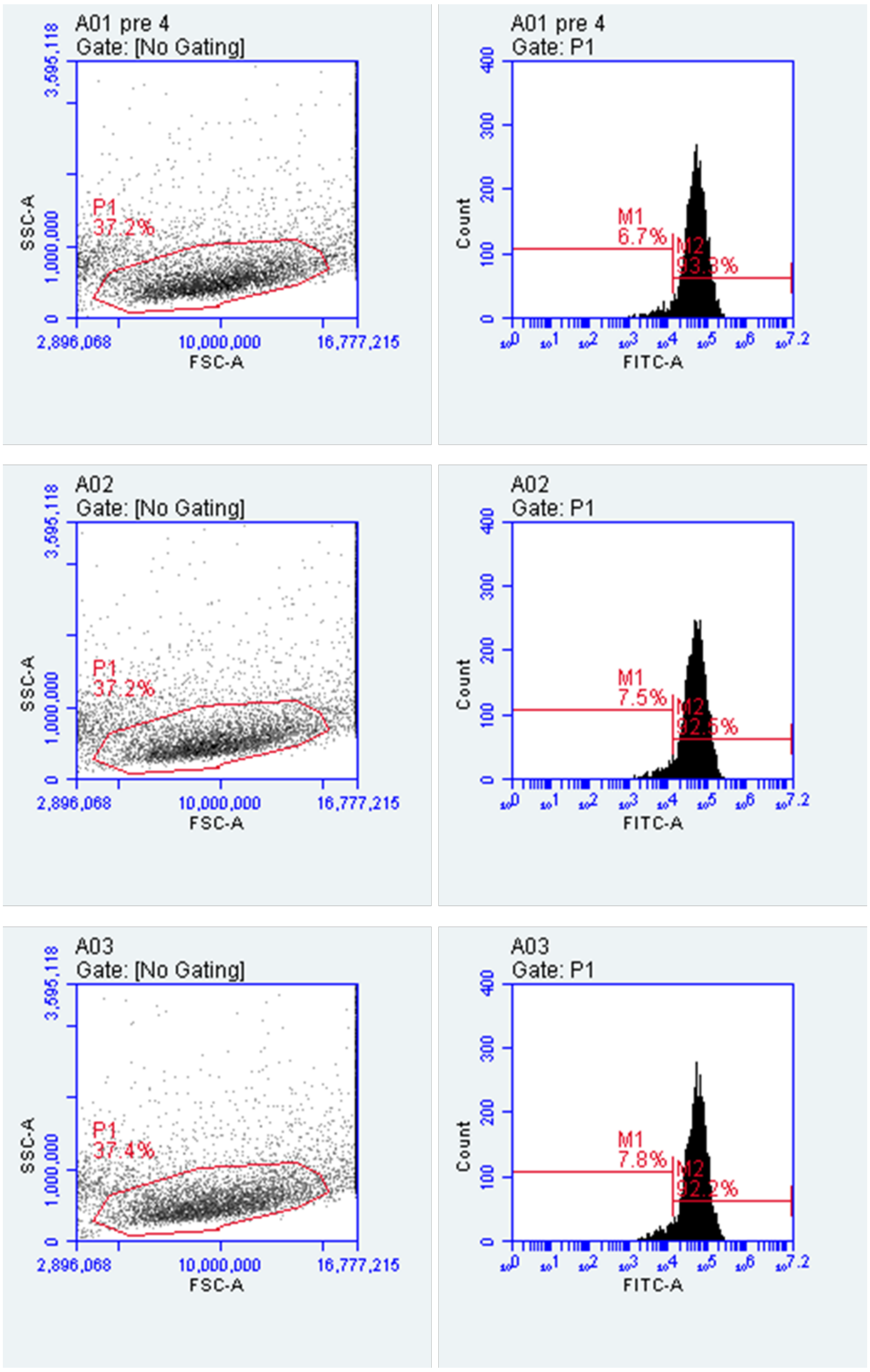
A01 pre 4
Gate: [No Gating]
SSC-A
FSC-A
P1
37.2%
A01 pre 4
Gate: P1
Count
FITC-A
M1
6.7%
M2
93.3%
A02
Gate: [No Gating]
P1
37.2%
A02
Gate: P1
M1
7.5%
M2
92.5%
A03
Gate: [No Gating]
P1
37.4%
A03
Gate: P1
M1
7.8%
M2
92.2%
2,896,068
10,000,000
16,777,215
3,595,118
1,000,000

(d) **F-1 37 °C**

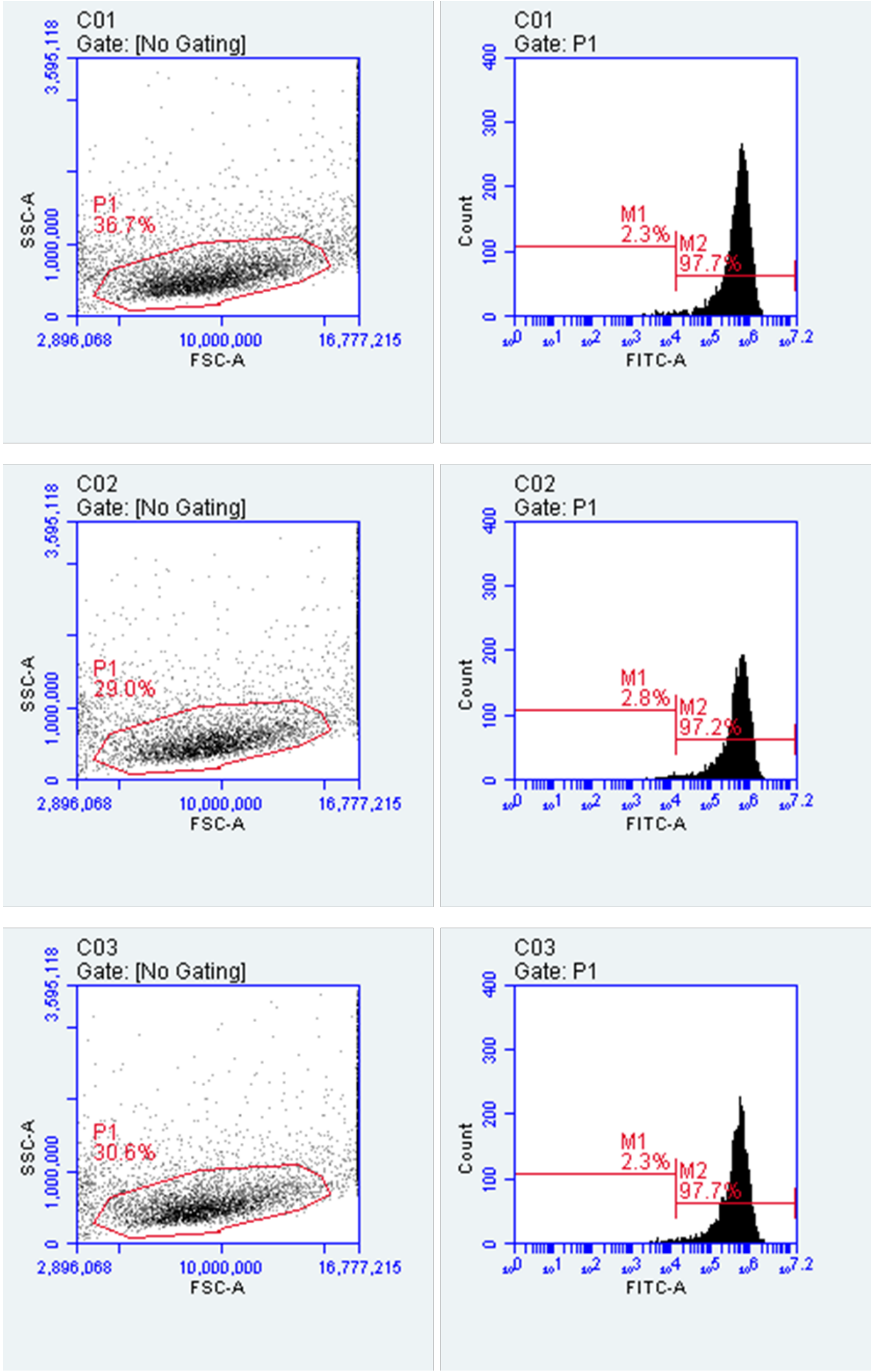
C01
Gate: [No Gating]
SSC-A
FSC-A
P1
36.7%
C01
Gate: P1
Count
FITC-A
M1
2.3%
M2
97.7%
C02
Gate: [No Gating]
SSC-A
FSC-A
P1
29.0%
C02
Gate: P1
Count
FITC-A
M1
2.8%
M2
97.2%
C03
Gate: [No Gating]
SSC-A
FSC-A
P1
30.6%
C03
Gate: P1
Count
FITC-A
M1
2.3%
M2
97.7%

(e) **F-3 4 °C**

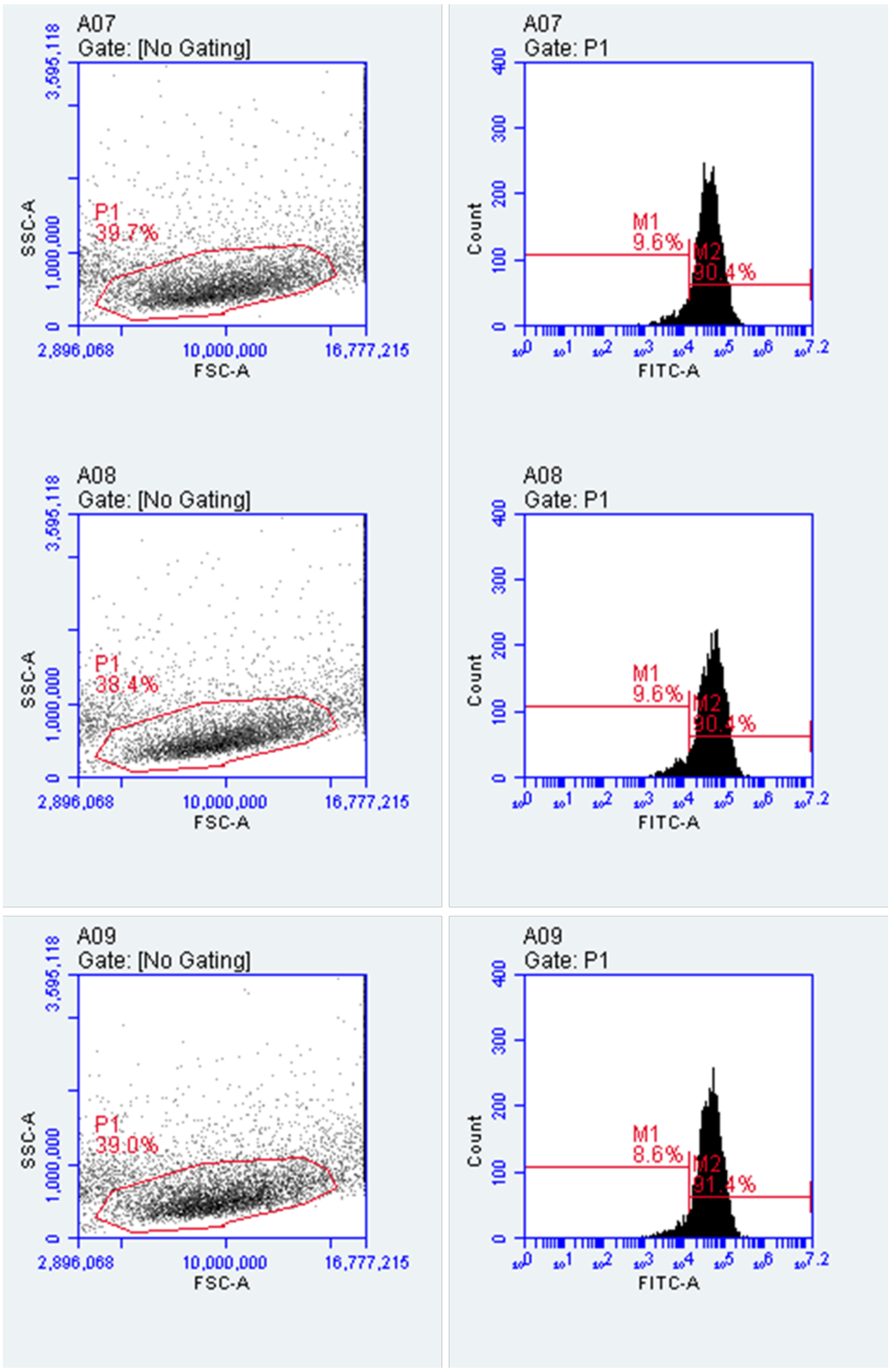
A07
Gate: [No Gating]
P1
39.7%
SSC-A
3,595,118
1,000,000
0
2,896,068
10,000,000
16,777,215
FSC-A
A07
Gate: P1
M1
9.6%
M2
90.4%
Count
400
300
200
100
0
FITC-A
A08
Gate: [No Gating]
P1
38.4%
SSC-A
3,595,118
1,000,000
0
2,896,068
10,000,000
16,777,215
FSC-A
A08
Gate: P1
M1
9.6%
M2
90.4%
Count
400
300
200
100
0
FITC-A
A09
Gate: [No Gating]
P1
39.0%
SSC-A
3,595,118
1,000,000
0
2,896,068
10,000,000
16,777,215
FSC-A
A09
Gate: P1
M1
8.6%
M2
91.4%
Count
400
300
200
100
0
FITC-A

(f) **F-3 37 °C**

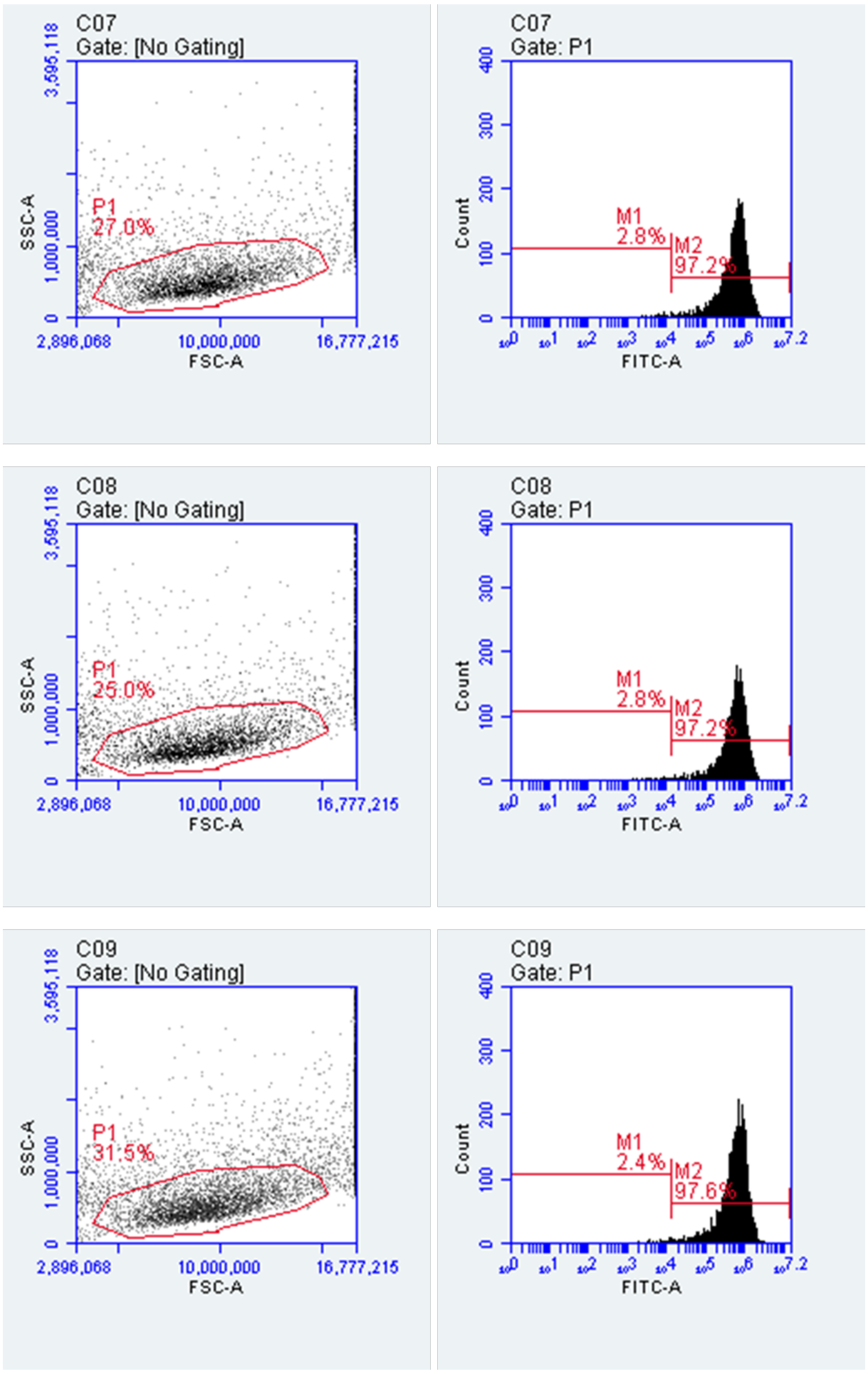

Figure S5. Raw data for scatter plots and histograms in flow cytometry in Figure 4b. These data showed treatment of each peptide at 4 or 37 °C (a-f). Each data set indicates n=3.

(a) **F-1**

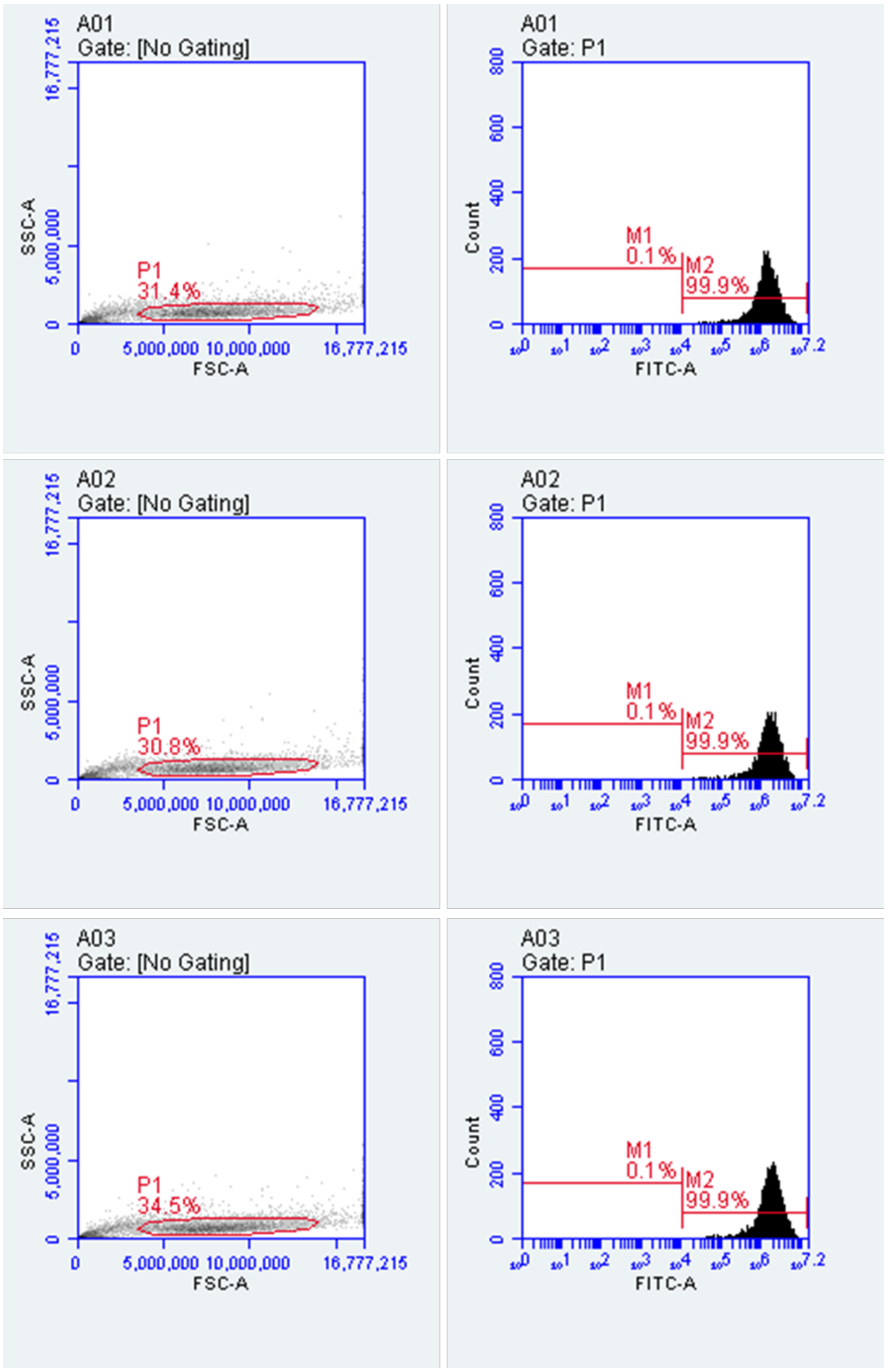
A01
Gate: [No Gating]
P1
31.4%
A01
Gate: P1
M1
0.1%
M2
99.9%
A02
Gate: [No Gating]
P1
30.8%
A02
Gate: P1
M1
0.1%
M2
99.9%
A03
Gate: [No Gating]
P1
34.5%
A03
Gate: P1
M1
0.1%
M2
99.9%
SSC-A
FSC-A
Count
FITC-A
0
5,000,000
10,000,000
16,777,215
200
400
600
800

(b) **F-2**

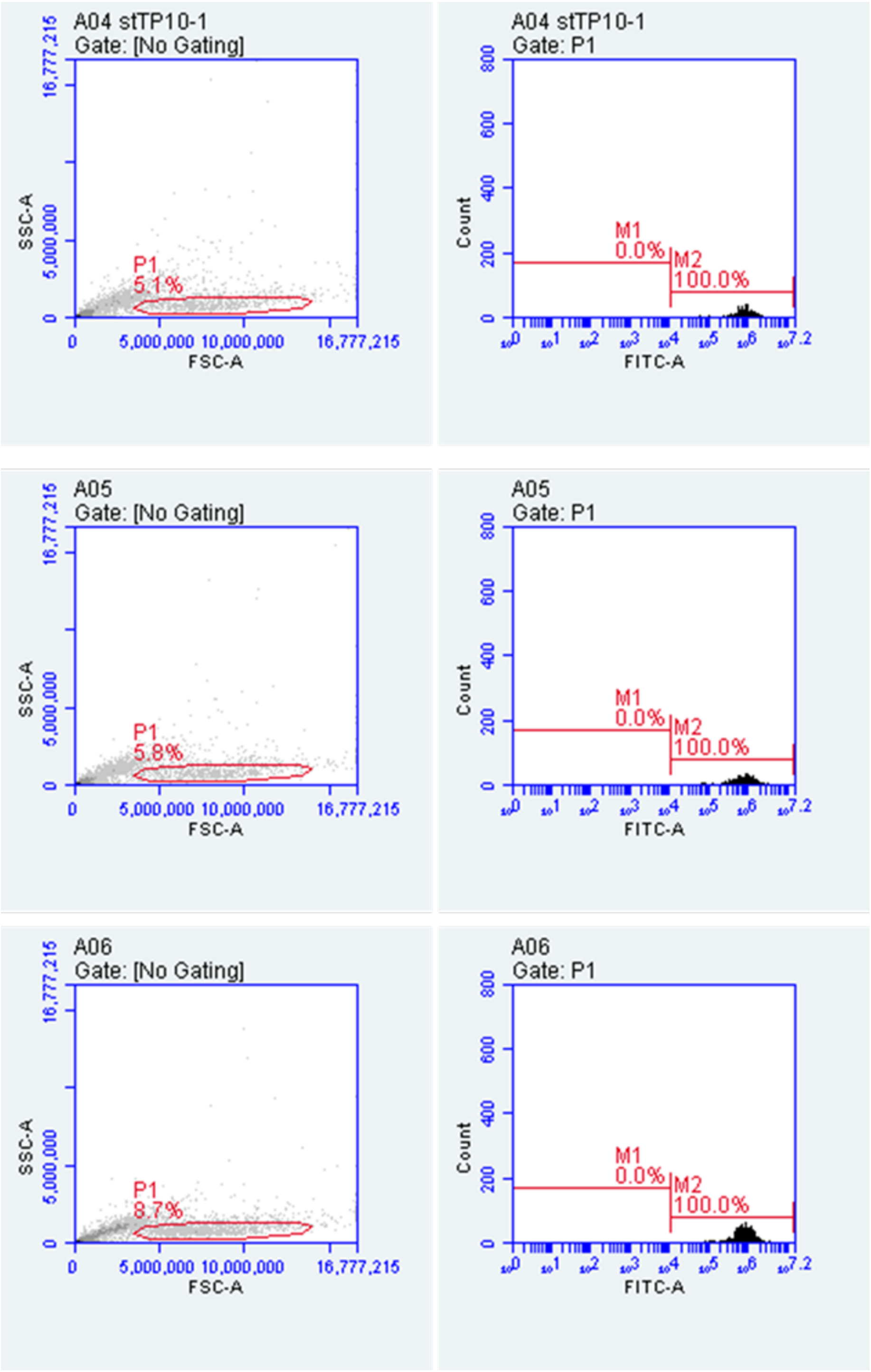

A04 stTP10-1
Gate: [No Gating]
SSC-A
FSC-A
P1
5.1%
A04 stTP10-1
Gate: P1
Count
FITC-A
M1
0.0%
M2
100.0%
A05
Gate: [No Gating]
SSC-A
FSC-A
P1
5.8%
A05
Gate: P1
Count
FITC-A
M1
0.0%
M2
100.0%
A06
Gate: [No Gating]
SSC-A
FSC-A
P1
8.7%
A06
Gate: P1
Count
FITC-A
M1
0.0%
M2
100.0%

(c) **F-3**

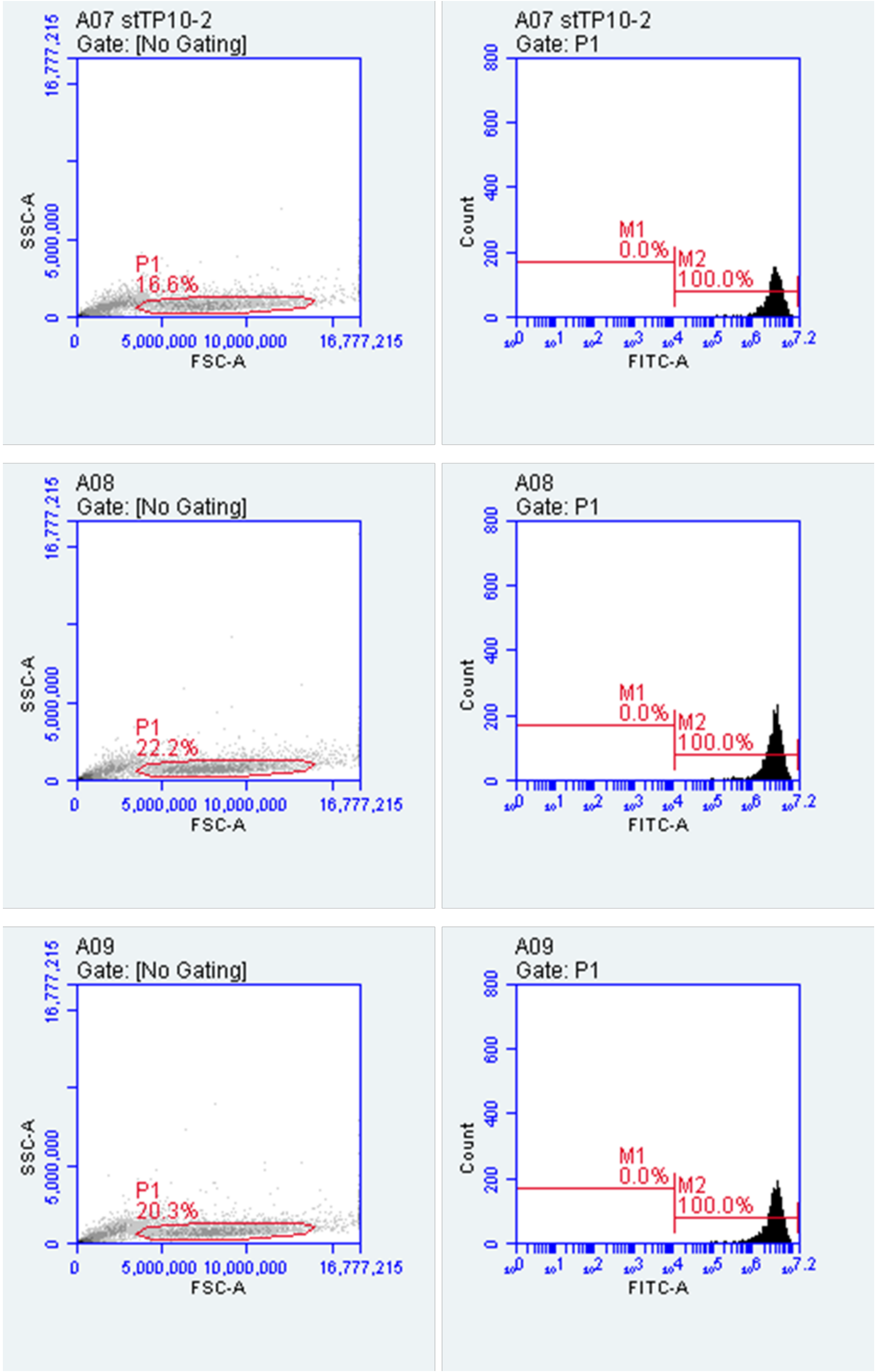
A07 stTP10-2
Gate: [No Gating]
P1
16.6%
SSC-A
FSC-A
A07 stTP10-2
Gate: P1
M1
0.0%
M2
100.0%
Count
FITC-A
A08
Gate: [No Gating]
P1
22.2%
SSC-A
FSC-A
A08
Gate: P1
M1
0.0%
M2
100.0%
Count
FITC-A
A09
Gate: [No Gating]
P1
20.3%
SSC-A
FSC-A
A09
Gate: P1
M1
0.0%
M2
100.0%
Count
FITC-A

(d) **F-4**

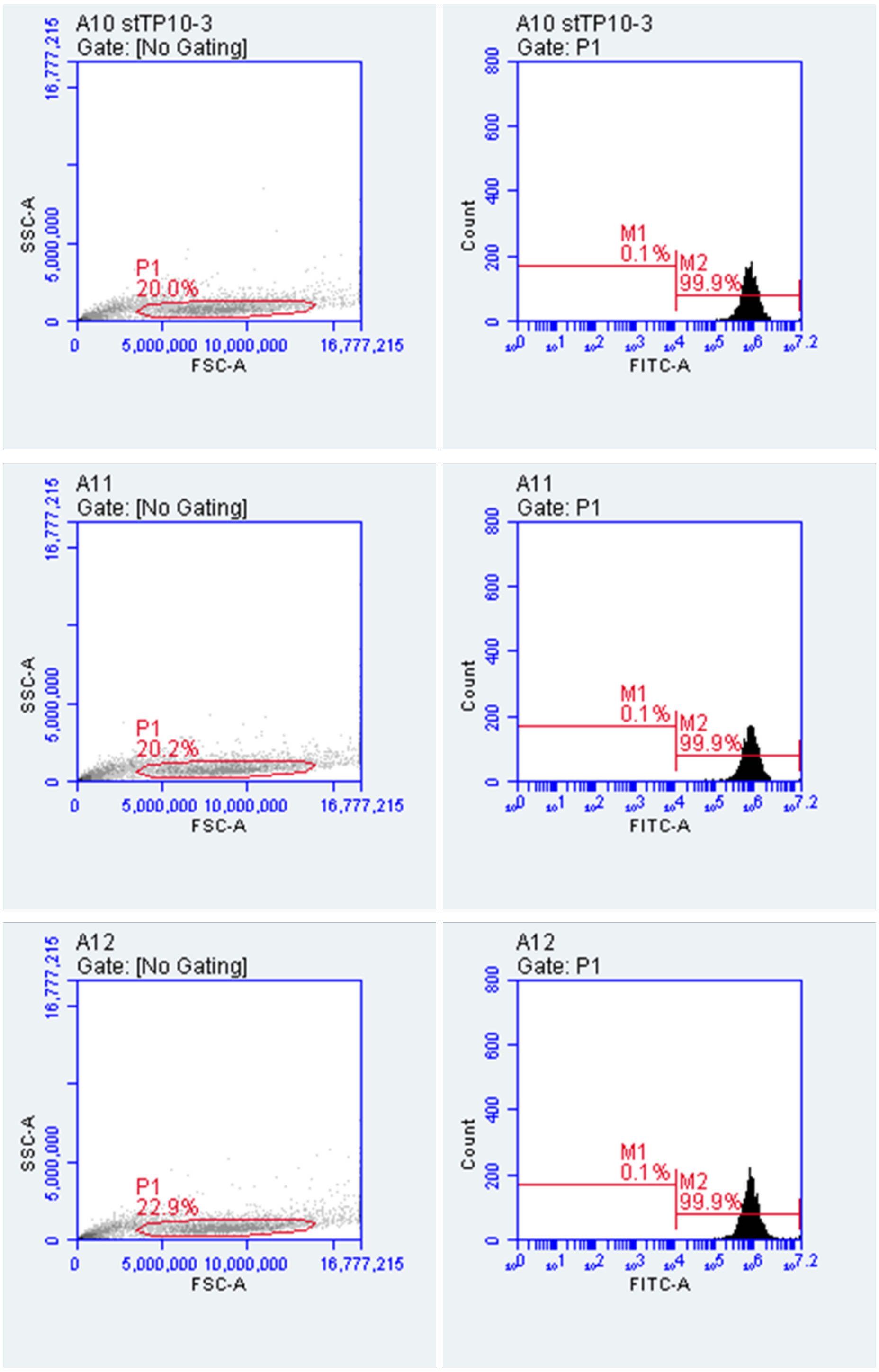
A10 stTP10-3
Gate: [No Gating]
P1
20.0%
SSC-A
FSC-A
0
5,000,000
10,000,000
16,777,215
A10 stTP10-3
Gate: P1
M1
0.1%
M2
99.9%
Count
FITC-A
A11
Gate: [No Gating]
P1
20.2%
SSC-A
FSC-A
A11
Gate: P1
M1
0.1%
M2
99.9%
Count
FITC-A
A12
Gate: [No Gating]
P1
22.9%
SSC-A
FSC-A
A12
Gate: P1
M1
0.1%
M2
99.9%
Count
FITC-A

(e) **JetPEI**

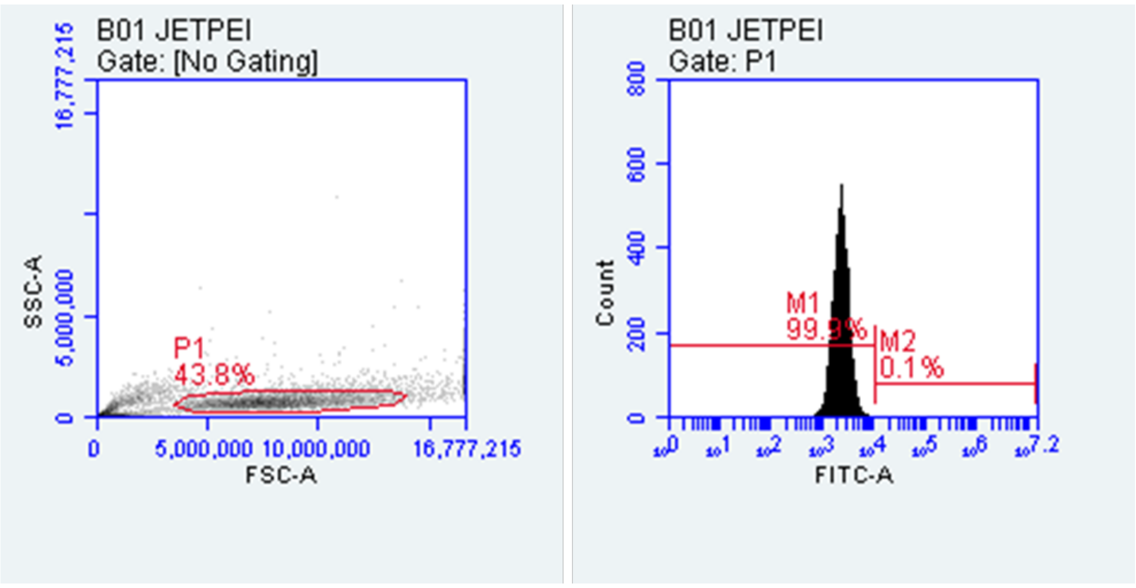
B01 JETPEI
Gate: [No Gating]
SSC-A
0
5,000,000
16,777,215
P1
43.8%
0
5,000,000 10,000,000
16,777,215
FSC-A
B01 JETPEI
Gate: P1
Count
0
200
400
600
800
M1
99.9%
M2
0.1%
FITC-A

(f) **control**

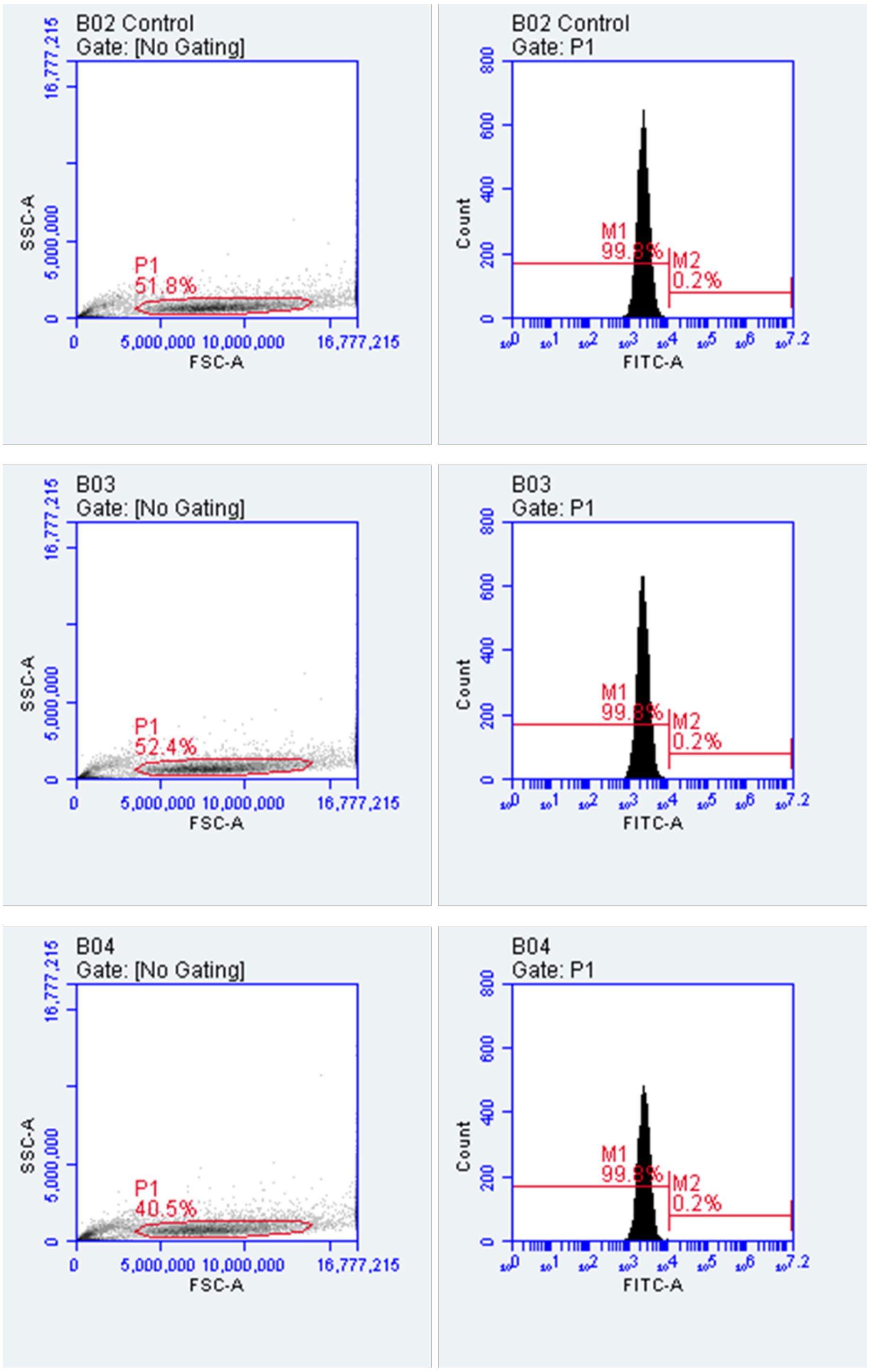

Figure S6. Raw data for scatter plots and histograms in flow cytometry in Figure 5. These data showed treatments of peptide/Cy5-pDNA complexes (a–d). (e) and (f) indicated positive and negative controls respectively.
